# Effector‐Mediated Spatial Reprogramming of Glycolate Oxidase Subverts Peroxisomal and Membrane‐Associated ROS Defences

**DOI:** 10.1111/pbi.70457

**Published:** 2025-11-12

**Authors:** Junjian Situ, Zijing Zhang, Yi Shao, Jiaying Feng, Feiteng Zhong, Muran Xiong, Wen Li, Peng Li, Xiaofan Zhou, Guibing Hu, Jietang Zhao, Minhui Li, Pinggen Xi, Xinxiang Peng, Zide Jiang, Guanghui Kong

**Affiliations:** ^1^ National Key Laboratory of Green Pesticide/Guangdong Province Key Laboratory of Microbial Signals and Disease Control South China Agricultural University Guangzhou China; ^2^ State Key Laboratory for Conservation and Utilization of Subtropical Agro‐Bioresources/Key Laboratory of Biology and Genetic Improvement of Horticultural Crops (South China), Ministry of Agriculture and Rural Affairs/Guangdong Litchi Engineering Research Center, College of Horticulture South China Agricultural University Guangzhou China; ^3^ State Key Laboratory for Conservation and Utilization of Subtropical Agro‐Bioresources/College of Life Sciences South China Agricultural University Guangzhou China

**Keywords:** litchi, *Peronophythora litchii*, photorespiration, ROS homeostasis, RXLR effector

## Abstract

Oomycete pathogens secrete hundreds of RXLR effectors into plant cells to modulate host immunity by targeting diverse plant proteins. Here, we report that the *Peronophythora litchii* RXLR effector PlAvh133 acts as a virulence factor and targets the litchi glycolate oxidase (GLO) LcGLO1, a key enzyme in photorespiration, thereby suppressing plant immunity. PlAvh133 localises to the plasma membrane (PM) in planta, and its first α‐helix is vital for both its LcGLO1‐binding activity and proper PM localisation. LcGLO1 is mainly confined to the peroxisomes, and its overexpression significantly enhanced resistance to downy blight in litchi. Conversely, silencing the *Nicotiana benthamiana* homologue of *LcGLO1* increases plant susceptibility to the oomycete pathogen. Critically, PlAvh133 causes the relocation of LcGLO1 from peroxisomes to the PM and inhibits its enzymatic activity, leading to increased plant susceptibility. PM‐localised LcGLO1 cooperates with catalase (CAT) LcCATB to suppress reactive oxygen species (ROS) burst. Meanwhile, PM‐localised LcGLO1 destabilises respiratory burst oxidase homologue (RBOH) LcRBOHD by interacting with calcium‐dependent protein kinase (CPK) LcCPK5, further reducing ROS production. Taken together, our findings unveil an unprecedented virulence mechanism by which a pathogen effector relocalises and inhibits host GLO1 activity, thereby simultaneously diminishing ROS production from both the peroxisomes and PM‐localised RBOHD.

## 1 Introduction

In nature, plants are constantly attacked by various pathogenic microbes, such as bacteria, fungi, and oomycetes. To successfully colonise their hosts, plant pathogens secrete a complex repertoire of effectors into plant cells or the extracellular space (Xu et al. 2021; Li, Wang, et al. 2023; Situ et al. 2024). Among them, RXLR effectors are the largest group of intracellular effectors found only in oomycete pathogens and defined by a conserved Arg‐any amino acid‐Leu‐Arg (RXLR) motif at their N‐terminus (Rehmany et al. 2005; Jiang et al. 2008). Thus far, all the Avirulence (Avr) proteins found in oomycete pathogens belong to this family (Wang et al. 2019). Mounting evidence has shown that RXLR effectors exploit multiple mechanisms to modulate host immunity to favour infection. For example, RNA silencing (RNAi), which plays a well‐established role in plant immunity, is suppressed by RXLR effectors from *Phytophthora sojae*, the causal agent of soybean stem and root rot (Qiao et al. 2013; Gui et al. 2022). Mitogen‐activated protein kinase (MAPK) signalling components have been shown to be targeted by several potato late blight pathogen *P. infestans* effectors (King et al. 2014; Murphy et al. 2018; Du et al. 2021). Gene expression is another key process interfered with by RXLR effectors (Huang et al. 2017; Kong et al. 2017; Qiu et al. 2022). Additionally, plant hormone pathways (Yang, Li, et al. 2018; Zhao et al. 2022), vesicle trafficking (Petre et al. 2021), autophagy (Dagdas et al. 2016), and endoplasmic reticulum (ER)‐stress‐mediated immunity (Fan et al. 2018) are also modulated by this kind of effector.

Photorespiration, a byproduct pathway of oxygenic photosynthesis that spans multiple cellular compartments and links primary metabolism, plays important roles in various biological processes (Bauwe et al. 2012; Jiang et al. 2023). In planta, glycolate oxidase (GOX/GLO) is a key enzyme in photorespiration, catalysing the conversion of glycolate into glyoxylate in peroxisomes with the production of H_2_O_2_ (Zhang et al. 2017; Corpas et al. 2020; Huang et al. 2025), which is independent of the H_2_O_2_‐producing enzyme, NADPH oxidase. Both glyoxylate and H_2_O_2_ are involved in plant disease resistance. Metabolites of glyoxylate, such as oxalate, glycine, and serine, are known to be implicated in defence responses in plants (Yu et al. 2010; Williams et al. 2011; Misra et al. 2016). As one kind of reactive oxygen species (ROS), H_2_O_2_ is associated with plant defence responses (Mignolet‐Spruyt et al. 2016; Noctor et al. 2018). Although the roles of glycolate oxidase in plant metabolism and resistance to abiotic stress conditions have been extensively studied, research on disease resistance is relatively limited (Xu et al. 2009; Chen et al. 2018; Ziotti et al. 2019). *GOX*‐silenced *Nicotiana benthamiana* and *Arabidopsis thaliana* *GOX* T‐DNA insertion mutants are compromised for nonhost resistance (Rojas et al. 2012). *A. thaliana gox* mutants have lower H_2_O_2_ accumulation, reduced callose deposition, and reduced electrolyte leakage upon inoculation with hypersensitive response‐causing nonhost pathogens. Silencing of *NbHAOX8*, *NbGOX1* and *NbGOX4* differently affected resistance to *Tobacco rattle virus*, *Xanthomonas oryzae* pv. oryzae (*Xoo*) and *Sclerotinia sclerotiorum* (Xu et al. 2018). In *Arabidopsis* plants, overexpressing glycolate oxidase (GO5) in chloroplasts resulted in increased levels of H_2_O_2_ and improved resistance toward the hemibiotrophic fungus *Colletotrichum higginsianum* (Schmidt et al. 2019). On the other hand, *Barley Stripe Mosaic Virus* γb protein was reported to bind with *N. benthamiana* glycolate oxidase and inhibit peroxisomal ROS production to promote viral infection (Yang, Wang, et al. 2018). The *P. infestans* effector Pi05910 suppresses and destabilises potato glycolate oxidase StGOX4 to promote plant susceptibility (Zhang et al. 2024). These reports provide a glimpse into the importance of glycolate oxidase in plant‐pathogen interactions. However, the specific mechanisms by which pathogen effectors target and modularise plant glycolate oxidase(s) to manipulate host processes remain largely unexplored.

A rapid H_2_O_2_ accumulation, also known as the oxidative burst, provides a direct antimicrobial effect and induces local and systemic signal molecules that are involved in the activation of defence responses (Mittler et al. 2022). Nevertheless, excessive accumulation of H_2_O_2_ can also be toxic to cells. Therefore, cellular homeostasis is characterised by a baseline level of H_2_O_2_, which is regulated by the balance between its cellular generation and its scavenging rates. Catalase (CAT), one of the major ROS scavenging enzymes, provides cells with a highly efficient mechanism for detoxifying H_2_O_2_. The function of this H_2_O_2_‐scavenging enzyme in immune responses has been investigated extensively (Jiang et al. 2023; Qiao et al. 2025). *A. thaliana* and tobacco CAT‐deficient mutants show H_2_O_2_ and salicylic acid (SA) accumulation, induced expression of *PR1* (pathogenesis‐related 1), a SA‐pathway marker gene, along with cell death (Takahashi et al. 1997; Mittler et al. 1999; Chaouch and Noctor 2010). The *A. thaliana* CAT2 functions as an H_2_O_2_ scavenger to help cells tolerate H_2_O_2_‐induced oxidative stress, and this activity is suppressed by the *Ralstonia solanacearum* effector RipAK (Sun et al. 2017). GLO has been demonstrated to physically interact with catalase in rice leaves, and this interaction can be deregulated by salicylic acid (SA) (Zhang et al. 2016). SA‐induced H_2_O_2_ accumulation results from SA‐induced GLO‐CAT dissociation (Zhang et al. 2016). Thus, the level of photorespiratory H_2_O_2_ is modulated by CATs, which may act as a hub in coordinating defence responses.

In plants, the NADPH oxidase respiratory burst oxidase homologue D (RBOHD), a membrane‐localised protein containing six conserved transmembrane domains in the middle with its N and C termini in the intracellular cytosol, produces apoplastic ROS and is involved in several pathways related to growth, development, and stress response (Liu et al. 2024). Plants recognise pathogens due to pathogen‐associated molecular patterns (PAMP) or secretory effector proteins that lead to the activation of RBOHD, which is a convergence point of pattern‐triggered immunity and effector‐triggered immunity signalling pathways. Previous research has documented that phosphorylation plays a crucial role in regulating RBOHD activity and stability (Kadota et al. 2014; Kimura et al. 2020; Lee et al. 2020). In Arabidopsis, the calcium‐dependent protein kinase 5 (CPK5) phosphorylates RBOHD, which occurs upon both PAMP and ROS stimulation. The rapid CPK5‐dependent defence reactions at distal sites are compromised in *cpk5* and *rbohd* mutants, supporting a model wherein CPK5 and RBOHD form a self‐propagating mutual activation circuit that facilitates ROS‐mediated cell‐to‐cell communication and rapid signal propagation, which is essential for activating defence responses at distant locations within the plant (Dubiella et al. 2013).

The hemibiotrophic oomycete pathogen *Peronophythora litchii* is the causal agent of litchi downy blight, which accounts for 30% of annual yield losses in production regions (Situ et al. 2024; Li et al. 2024). During infection, *P. litchii* secretes a repertoire of RXLR effectors into host cells to interfere with host immunity (Ye et al. 2016; Situ et al. 2020; Li, Li, et al. 2023). In this study, we found that PlAvh133 was a virulence factor that promoted infection by *P. litchii*. During the *P. litchii* infection process, PlAvh133 targets the litchi glycolate oxidase 1 (LcGLO1), causing LcGLO1 to relocate to the plasma membrane (PM) and a decrease in enzymatic activity. On the PM, LcGLO1 suppresses the ROS burst by binding with LcCATB and LcCPK5. The binding of PM‐localised LcGLO1 with LcCPK5 decreases the protein stability of LcRBOHD. Our study reveals a novel strategy exploited by a phytopathogenic oomycete to manipulate the plant immune system.

## 2 Results

### 2.1 PlAvh133 Is a Virulence Factor of *P. Litchii*

**FIGURE 1:**
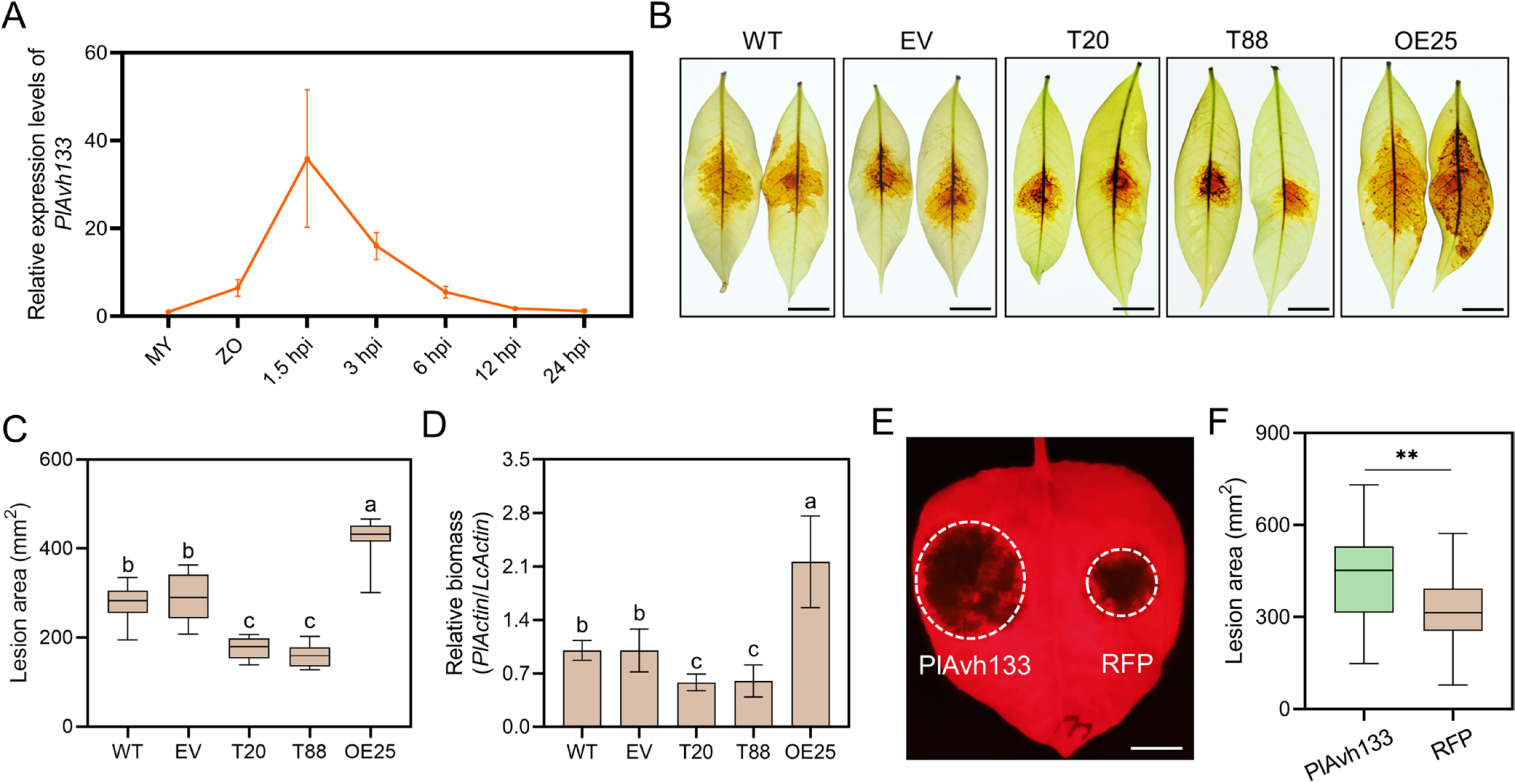
PlAvh133 is required for the virulence of *Peronophythora litchii*. (A) *PlAvh133* was induced in zoospores and early infection stages. qRT‐PCR was employed to assess the relative transcript levels of *PlAvh133* in different developmental and infection stages. MY, *P. litchii* mycelia grown on CA; ZO, zoospores. Litchi leaves inoculated with *P. litchii* zoospores were harvested at 1.5, 3, 6, 12, and 24 hpi. The relative expression levels were calibrated to MY set as 1. The constitutively expressed gene, *PlActin*, was used as an internal reference. (B–D) *P. litchii PlAvh133* knockout mutants exhibited reduced virulence. Tender litchi leaves were inoculated with zoospore suspensions from *P. litchii* wild type (WT) strain SHS3, a control strain transformed by the empty vector (EV), *PlAvh133* knockout mutants (T20 and T88), or an overexpression strain (OE25). Disease symptoms were photographed at 48 hpi, and the lesion areas were measured to evaluate infection severity. Scale bar, 1 cm. Relative biomass of *P. litchii* was determined by qPCR. Data represent means ± SD. Experiments were repeated three times with similar results. Different letters indicate significant differences using Duncan's multiple range test at *p* < 0.01; *n* = 10. (E, F) Expression of PlAvh133 in *N. benthamiana* enhanced susceptibility to *P. capsici*. *N. benthamiana* leaves expressing PlAvh133‐RFP or RFP were inoculated with *P. capsici*. Lesion development was measured and photographed at 48 hpi. Scale bar, 1 cm. Asterisks indicate significant differences. **p < 0.01; Student's *t*‐test; *n* = 46. In C and F, the central horizontal line denotes the median; vertical box height corresponds to the interquartile range; and the whiskers show the maximum and minimum values within the analysed dataset.

To understand *P. litchii* pathogenesis, we analysed the time‐course RNA‐seq data from the infection stages to identify highly up‐regulated RXLR effector genes. As *PlAvh133* is one of the most highly expressed RXLR‐encoding genes in early infection stages (Figure 1A), we speculated that PlAvh133 may play a crucial role during the plant‐pathogen interaction. To determine the function of *PlAvh133* in the virulence of *P. litchii*, we deleted *PlAvh133* from the *P. litchii* genome by the CRISPR‐mediated genome editing method, and two independent disrupted mutants of *PlAvh133* (T20 and T88) were obtained (Figure S1A–C). To determine the virulence of the *PlAvh133* mutants, we performed an infection assay on tender litchi leaves. The results showed that the lesion area caused by *PlAvh133* knockout mutants was significantly reduced compared to either the wild type strain SHS3 or the empty vector control (EV; Figure 1B,C). To assess infection more precisely, we used qPCR to measure the relative pathogen biomass (the ratio of *P. litchii* DNA to litchi DNA) in infected tissues. The biomass of T20 or T88 in the infected plant tissues was significantly lower than that of WT or EV strains (Figure 1D). Furthermore, a *PlAvh133* overexpression strain was obtained, and the infection assay revealed that overexpression significantly promoted pathogen infection (Figure 1B–D and Figure S1C). All of the above strains had similar growth rates and cultural morphology as the wild type (WT; Figure S1D,E).

To further investigate whether PlAvh133 is a virulence effector that promotes pathogen colonisation in planta, PlAvh133‐RFP and the RFP control were transiently expressed in *N. benthamiana* leaves and subsequently used for infection assays. The results demonstrated that compared to the RFP control, expression of PlAvh133 significantly promoted *Phytophthora capsici* colonisation at 48 h post‐inoculation (hpi; Figure 1E,F). The expression of PlAvh133 was confirmed by Western blot (Figure S1F). We obtained similar results when a GFP tag was fused to the N‐terminus of PlAvh133 (Figure S1G–I). Taken together, these results indicated that *PlAvh133* is crucial for the pathogenicity of *P. litchii*.

### 2.2 PlAvh133 Interacts With Litchi GLO1 and the First α‐Helix of PlAvh133 Is Vital for Its LcGLO1‐Binding Activity and Plasma Membrane Localisation

**FIGURE 2:**
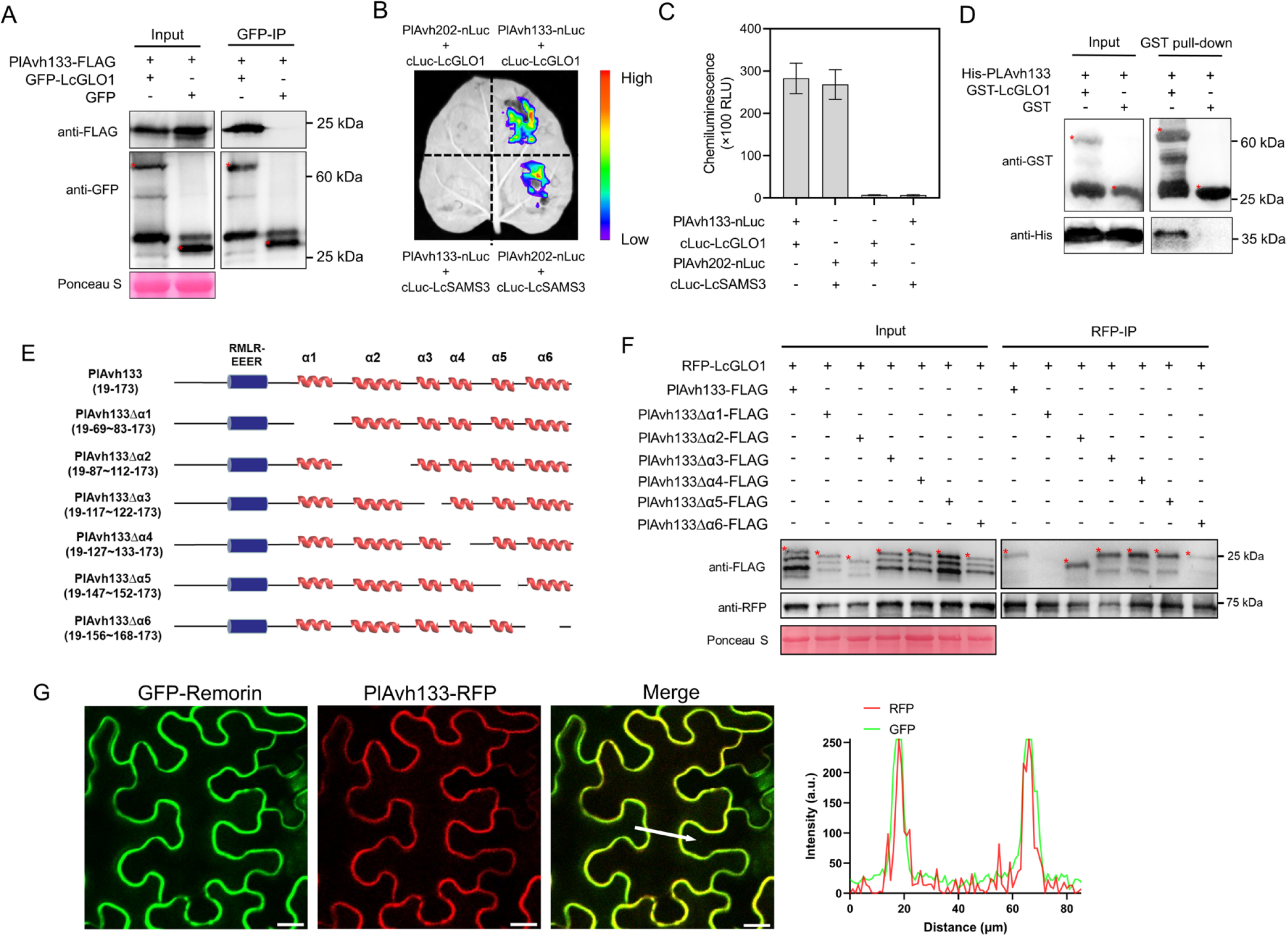
PlAvh133 interacts with LcGLO1 and the first α‐helix of PlAvh133 is vital for its LcGLO1‐binding activity. (A) In vivo co‐IP of PlAvh133 with LcGLO1. Total proteins were extracted from *N. benthamiana* leaves expressing GFP‐LcGLO1 or GFP (control) together with PlAvh133‐FLAG. The immune complexes were pulled down using anti‐GFP agarose beads. Protein loading is indicated by Ponceau S staining. (B, C) Interaction of PlAvh133 with LcGLO1 detected by split luciferase complementation (SLC). Luciferase (LUC) was divided into C‐ and N‐termini, with PlAvh133 fused to the N‐terminus of LUC and LcGLO1 fused to the C‐terminus. Fluorescence intensity and LUC activity were observed and measured at 48 h post‐agroinfiltration (hpa). (D) Confirmation of the interaction between PlAvh133 and LcGLO1 by GST pull‐down. His‐PlAvh133 and GST‐LcGLO1 were expressed in *E. coli* . Co‐precipitation of His‐PlAvh133 with GST‐LcGLO1 was examined by Western blotting before (input) and after affinity purification (pull‐down) using glutathione agarose beads. In Western blot assays, expected protein bands are indicated by red asterisks. All of the above experiments were performed three times with similar results. (E, F) Co‐IP assay between PlAvh133 deletion mutants and LcGLO1. The left panel is a schematic view of the PlAvh133 deletion mutants, and the right panel is the co‐IP assay between PlAvh133 deletion mutants and LcGLO1. Total proteins were extracted from *N. benthamiana* leaves expressing RFP‐LcGLO1 together with PlAvh133‐FLAG derivatives. The immune complexes were pulled down using anti‐RFP agarose beads. Protein loading is indicated by Ponceau S staining. Expected protein bands are indicated by red asterisks. (G) The subcellular localisation of PlAvh133 in *N. benthamiana* leaf cells. Fluorescence in epidermal cells was detected by confocal microscopy 48 hpa. Scale bars, 20 μm. The fluorescence intensity charts on the right correspond with the white arrow cross‐sections in the images to their left.

To further elucidate the mechanisms of PlAvh133 virulence function, we expressed GFP‐tagged PlAvh133 in *N. benthamiana* leaves, and then immunoprecipitated GFP‐PlAvh133 proteins followed by liquid chromatography–tandem mass spectrometry (LC–MS/MS) analyses. Among the detected *N. benthamiana* proteins (Table S1), a (S)‐2‐hydroxy‐acid oxidase (also named glycolate oxidase, NbGLO1) was identified. We further searched the NbGLO1 homologues in the litchi database and identified a highly homologous protein, named LcGLO1 (Figure S2A,B). We performed co‐immunoprecipitation (co‐IP) assays to confirm the association between FLAG‐tagged PlAvh133 and GFP‐tagged LcGLO1 proteins in planta. The results showed that PlAvh133 interacted with LcGLO1, while no interaction was observed with the GFP control (Figure 2A). The co‐IP between PlAvh133 and NbGLO1 showed similar results (Figure S3). The luciferase complementation assay was also used to confirm the interaction between PlAvh133 and LcGLO1. PlAvh202 and LcSAMS3, which have been proven to interact with each other in previous studies (Li, Li, et al. 2023), were used as the positive control. Similar to the positive control, strong fluorescence intensity was observed when PlAvh133‐nluc co‐expressed with cluc‐LcGLO1 (Figure 2B,C). We then performed in vitro pull‐down assays to confirm the direct physical association between PlAvh133 and LcGLO1. Recombinant GST‐LcGLO1 and His‐PlAvh133 proteins were expressed in *Escherichia coli* . GST (as a negative control) and GST‐LcGLO1 were purified from a whole‐cell lysate using glutathione resins and then incubated with His‐PlAvh133. The His‐PlAvh133 proteins were specifically detected in GST‐LcGLO1 pull‐down products but not in the GST control (Figure 2D), suggesting that PlAvh133 interacted with LcGLO1 in vitro. Based on the protein secondary structure predicted by JPred4 (https://www.compbio.dundee.ac.uk/jpred/index.html), PlAvh133 contains six α‐helices downstream of the signal peptide (Figure 2E). To identify which domains are crucial for PlAvh133 binding with its target protein, a series of α‐helix deletion mutants were constructed (PlAvh133∆α1, PlAvh133∆α2, PlAvh133∆α3, PlAvh133∆α4, PlAvh133∆α5, and PlAvh133∆α6) and co‐IP assays were performed to assess their ability to associate with LcGLO1. As shown in Figure 2F, the α2, α3, α4, α5, and α6‐helix deletion mutants of PlAvh133 were successfully co‐purified with LcGLO1. However, the α1‐helix deletion mutant did not co‐immunoprecipitate with LcGLO1 (Figure 2F). To further identify the key interaction area within the α1‐helix, and to avoid any potential disruption of protein folding that could arise from simple residue deletions, we divided the 15 amino acids of the α1‐helix into three parts (α1‐helixM1, α1‐helixM2, and α1‐helixM3) and separately substituted each with alanine (Figure S4A). Co‐IP results demonstrated that all three mutants retained the ability to interact with LcGLO1 (Figure S4B). However, when the first 10 and the last 10 amino acids were individually replaced with alanine (resulting in α1‐helixM4 and α1‐helixM5, respectively), the α1‐helixM5 mutant was found to lose its binding capacity to LcGLO1 (Figure S4B). The entire alanine substitution of the α1‐helix (α1‐helixM6) also abolished the interaction with LcGLO1 (Figure S4B). Taken together, these results demonstrated that PlAvh133 interacts with LcGLO1 and the α1‐helix of PlAvh133 is required for their binding.

We next analysed the subcellular localisation of PlAvh133 and the structural basis of this localisation. The wild type PlAvh133 fused with C‐terminal RFP was transiently expressed in *N. benthamiana*, and confocal imaging showed that wild type PlAvh133 substantially localised to the PM (Figure 2G). A Remorin protein with an N‐terminal GFP fusion, which is a PM‐localised protein, was used as a PM marker (Bozkurt et al. 2014). The expression of PlAvh133‐RFP and GFP‐Remorin was confirmed by Western blot (Figure S5A). To confirm this result, PlAvh133 fused to an N‐terminal GFP (GFP‐PlAvh133) was also transiently expressed in *N. benthamiana*. Again, green fluorescence was observed at the PM (Figure S5B). Additionally, we examined the subcellular localisation of PlAvh133 after plasmolysis and found that it was predominantly localised to the PM (Figure S5C). To study the structural basis of PlAvh133 localisation, the six PlAvh133 α‐helices deletion mutants fused with C‐terminal RFP were transiently expressed in *N. benthamiana*. Confocal observation showed that deletion of any single α‐helix still allowed PM localisation (Figure S6A). However, apart from PM localisation, PlAvh133∆α1 showed nuclear localisation (Figure S6A). We further examined the subcellular localisation of the α1‐helixM5 and α1‐helixM6 mutants and observed that only the substitution of the entire α1‐helix with alanine, rather than the last 10 amino acids alone, resulted in a similar alteration in subcellular localisation (Figure S6B). All of these results indicated that the α1‐helix is important for proper PM localisation of PlAvh133.

### 2.3 PlAvh133 Causes LcGLO1 to Relocate to the PM When Co‐Expressed in Planta

**FIGURE 3:**
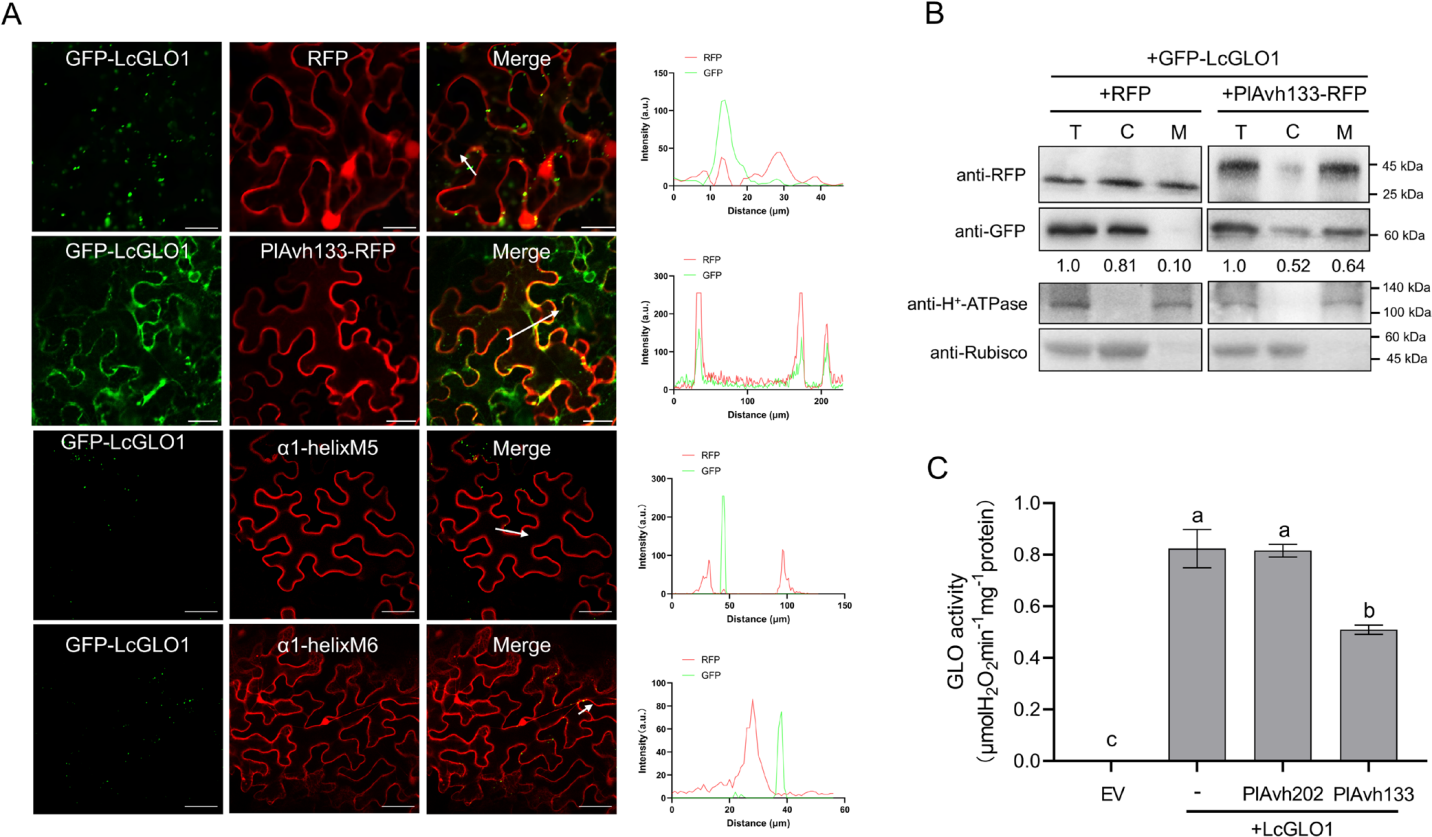
PlAvh133 causes relocation of LcGLO1 to the PM and inhibits its enzymatic activity. (A) Subcellular localisation of LcGLO1. Cells of *A. tumefasciens* harbouring GFP‐LcGLO1 were co‐infiltrated with *A. tumefasciens* harbouring RFP, PlAvh133‐RFP, α1‐helixM5‐RFP, or α1‐helixM6‐RFP into *N. benthamiana* leaves. Photographs were taken at 48 hpa. Scale bars, 20 μm. The fluorescence intensity charts on the right correspond respectively with the white arrow cross‐sections in the images to their left. (B) Subcellular fractionation of extracts from *N. benthamiana* leaves co‐expressing GFP‐LcGLO1 with PlAvh133‐RFP or RFP. C, cytosolic fraction; M, microsomal fraction; T, total protein. (C) PlAvh133 inhibits the enzymatic activity of the LcGLO1. The crude enzymes of His‐LcGLO1, His‐PlAvh133, and His‐PlAvh202 were extracted from *E. coli* strains BL21 expressing pET32a‐LcGLO1, pET32a‐PlAvh133, and pET32a‐PlAvh202, respectively. EV represents the crude enzyme extracted from *E. coli* strains BL21 expressing an empty vector. The data represent means ± SD of 3 independent experiments. Different letters indicate significant differences using Duncan's multiple range test at *p* < 0.01.

To evaluate the underlying mechanism of PlAvh133‐LcGLO1 interaction, we observed the localisation of PlAvh133 and LcGLO1 during transient co‐expression in *N. benthamiana*. When GFP‐LcGLO1 and RFP control were co‐expressed in *N. benthamiana*, LcGLO1 localised in the peroxisomes as expected (Figure 3A; Figure S7A). Interestingly, when GFP‐LcGLO1 was co‐expressed with PlAvh133‐RFP, LcGLO1 strongly localised in the PM (Figure 3A; Figure S7B). To test if direct interaction between LcGLO1 and PlAvh133 was required for the relocation of LcGLO1 to the PM, the LcGLO1‐non‐interacting mutant PlAvh133 α1‐helixM5 and α1‐helixM6 were tested for their ability to cause LcGLO1 to relocate. As shown in Figure 3A and Figure S7C, α1‐helixM5 and α1‐helixM6 could not cause LcGLO1 to relocate to the PM when they were transiently co‐expressed in *N. benthamiana*. To confirm this localisation pattern, we employed biochemical fractionation by transiently co‐expressing GFP‐LcGLO1 with PlAvh133‐RFP or RFP in *N. benthamiana*. As expected, when GFP‐LcGLO1 was co‐expressed with RFP, GFP‐LcGLO1 accumulated mainly in the cytosolic fraction. However, GFP‐LcGLO1 was enriched in microsomes, similar to H^+^‐ATPase, in the presence of PlAvh133 (Figure 3B). These results suggest that PlAvh133 causes LcGLO1 to relocate to the PM through a specific interaction.

### 2.4 PlAvh133 Inhibits LcGLO1 Enzymatic Activity

Although the canonical reaction site of GLO1 is the peroxisome, its substrate, glycolate, originates in the chloroplast and is exported via specific transporters (South et al. 2017). Consequently, even when GLO is not peroxisome‐localised it may still encounter glycolate and catalyse H_2_O_2_ production, although the distribution and concentration of glycolate across cellular compartments remain poorly characterised. Given that direct inhibition of target enzyme activity is a well‐documented virulence strategy of pathogen effectors (Cao, Zhang, et al. 2024), we next tested whether PlAvh133 impairs the enzymatic activity of LcGLO1. To investigate this possibility, we tested LcGLO1 enzymatic activity through in vitro assays. The results showed that LcGLO1 had strong enzymatic activity as expected (Figure 3C). While the addition of PlAvh133 greatly reduced LcGLO1 enzymatic activity. Besides, another *P. litchii* RXLR effector, PlAvh202, had no significant impact on the enzymatic activity of GLO1 (Figure 3C). These results demonstrated that PlAvh133 could also inhibit LcGLO1 function by reducing its enzymatic activity.

### 2.5 LcGLO1 Relocation to the PM Is Sufficient to Promote Disease Susceptibility

**FIGURE 4:**
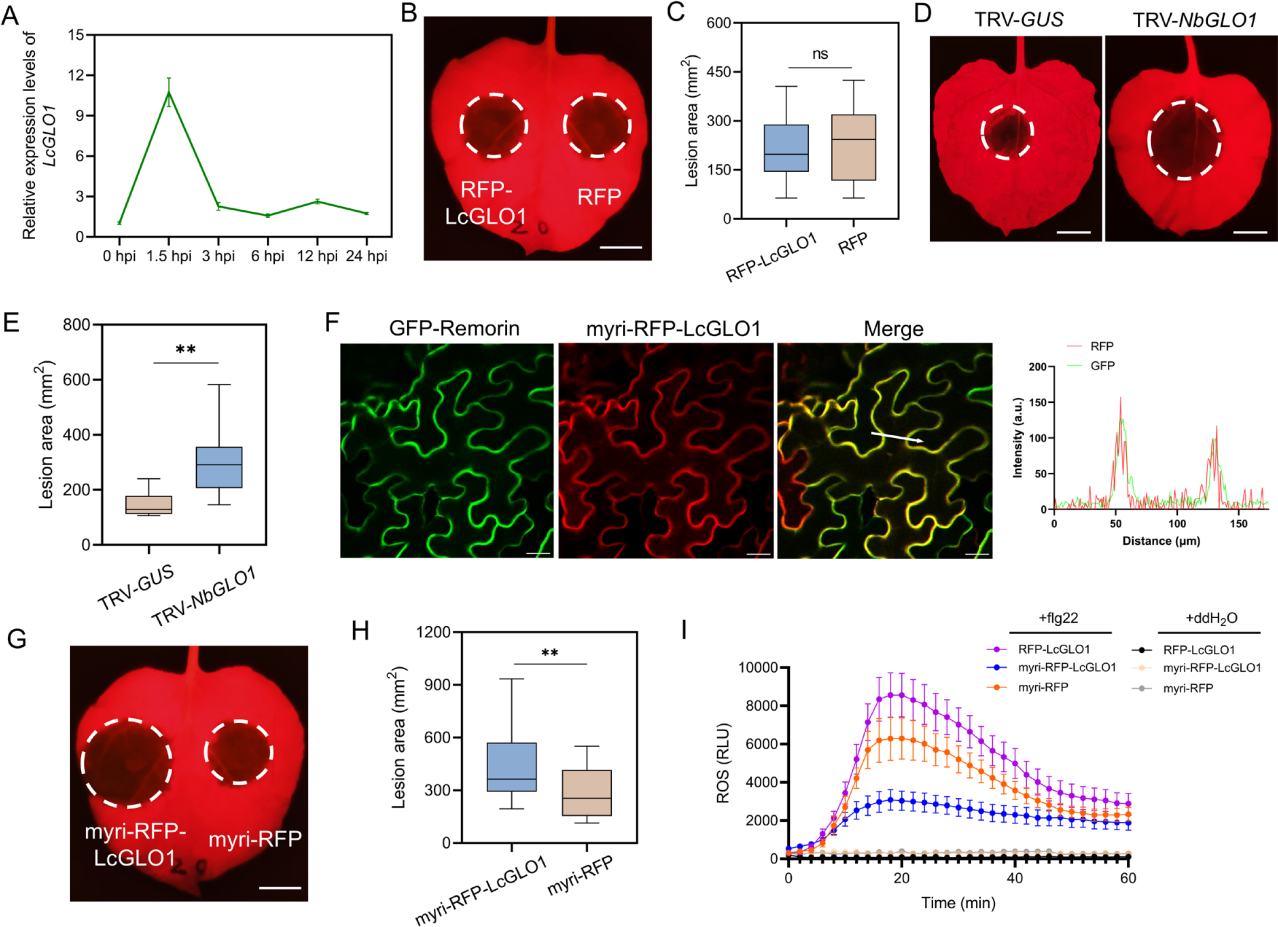
LcGLO1 relocation to the PM is sufficient to promote disease susceptibility. (A) Expression profile of *LcGLO1*. The relative transcript levels of *LcGLO1* during different infection stages were assessed by qRT‐PCR. Litchi leaves inoculated with *P. litchii* zoospores were harvested at 0, 1.5, 3, 6, 12, and 24 hpi. The relative expression level was normalised to the levels for non‐inoculated leaves. The constitutively expressed gene *LcActin* was used as an internal reference. Data represent means ± SD. Experiments were repeated three times with similar results. (B and C) Wild‐type LcGLO1 did not contribute to resistance against *P. capsici*. *N. benthamiana* leaves expressing RFP‐LcGLO1 or RFP were inoculated with *P. capsici*. Lesion development was measured and photographed at 48 hpi. Scale bar, 1 cm. (D and E) Silencing of *NbGLO1* enhances susceptibility to *P. capsici*. The lesions in *N. benthamiana* leaves were measured and photographed at 48 hpi. Scale bar, 1 cm. (F) Subcellular localisation of LcGLO1 tagged with the N‐terminal PM localisation signal myri. Cells of *A. tumefasciens* harbouring myri‐RFP‐LcGLO1 were co‐infiltrated with cells of *A. tumefasciens* harbouring GFP‐Remorin into *N. benthamiana* leaves. Photographs were taken at 48 hpa. Scale bars, 20 μm. The fluorescence intensity charts on the right correspond respectively with the white arrow cross‐sections in the images to their left. (G, H) PM‐localised LcGLO1 promoted susceptibility to *P. capsici*. *N. benthamiana* leaves expressing myri‐RFP‐LcGLO1 or myri‐RFP were inoculated with *P. capsici*. Lesion development was measured and photographed at 48 hpi. Scale bar, 1 cm. (I) ROS burst induced by flg22 in *N. benthamiana* leaves expressing RFP‐LcGLO1, myri‐RFP‐LcGLO1, and myri‐RFP. Relative luminescence units (RLU) indicate relative amounts of H_2_O_2_ production induced by 10 μM flg22 or ddH_2_O in leaf strips. The results shown are representative of three independent experiments. Each data point consists of 8 replicates. Error bars indicate SD. In C, E and H, the central horizontal line denotes the median; vertical box height corresponds to the interquartile range; and the whiskers show the maximum and minimum values within the analysed dataset. Asterisks indicate significant differences (**p < 0.01; ns, no significant difference; Student's *t*‐test; *n* ≥ 12).

To explore the possible contributions of LcGLO1 to litchi immunity, we first determined the expression pattern of *LcGLO1* during the infection stages. The results from qRT‐PCR showed that *LcGLO1* expression rapidly increased at 1.5 hpi (Figure 4A). To study the function of LcGLO1 in plant defence, we transiently expressed RFP‐LcGLO1 and the control RFP into the right and left panels of *N. benthamiana* leaves, and performed infection assays. The results showed that the lesion areas and pathogen biomass in LcGLO1 expression leaves were comparable to the control (Figure 4B,C; Figure S8A). The expression of LcGLO1 was confirmed by Western blot (Figure S8B). To further verify the function of the GLO1, the *N. benthamiana* homologue *NbGLO1* [Sol Genomics Network database (https://solgenomics.net/), gene ID: Niben101Scf04174g05001.1] was silenced via virus‐induced gene silencing (VIGS; Figure S8C). Subsequent infection assays revealed that silencing of *NbGLO1* significantly impaired resistance to the oomycete pathogen (Figure 4D,E). However, the expression of NbGLO1 showed an enhancement of disease resistance (Figure S8D,E). Because LcGLO1 relocates to the PM when PlAvh133 is present, this led to our hypothesis that LcGLO1 acts as a negative regulator of immunity when localised in the PM. Therefore, the myristoylation (myri) sequence, which functions as the PM localisation signal (Vazquez et al. 2023), was fused to the N‐terminus of RFP‐LcGLO1. Confocal observations and subcellular fractionation confirmed that myri‐RFP‐LcGLO1 localised to the PM (Figure 4F and Figure S8F–H). Subsequently, we transiently expressed myri‐RFP‐LcGLO1 in *N. benthamiana* leaves followed by an infection assay. Compared with leaves expressing the control myri‐RFP, the expression of myri‐RFP‐LcGLO1 significantly increased susceptibility to infection by *P. capsici* (Figure 4G,H; Figure S8I).

The homeostasis of ROS is tightly controlled by photorespiration (Jiang et al. 2023). Flg22 is a conserved peptide that can stimulate ROS burst as an early PTI response (Felix et al. 1999). Hence, we used this representative pathogen‐associated molecular pattern (PAMP) to evaluate the ROS response induced by LcGLO1. The results showed that compared to the control, the flg22‐induced ROS burst increased by approximately 36% in RFP‐LcGLO1‐expressing leaves (Figure 4I). By contrast, the myri‐RFP‐LcGLO1‐expressing leaves showed reduced flg22‐induced ROS burst to 42% (Figure 4I). Taken together, these results indicated that LcGLO1 localised to the PM is sufficient to reduce ROS accumulation and promote plant susceptibility to infection.

### 2.6 PM‐Localised LcGLO1‐LcCATB Complex Negatively Regulate Plant Immunity by Eliminating the ROS Burst

**FIGURE 5:**
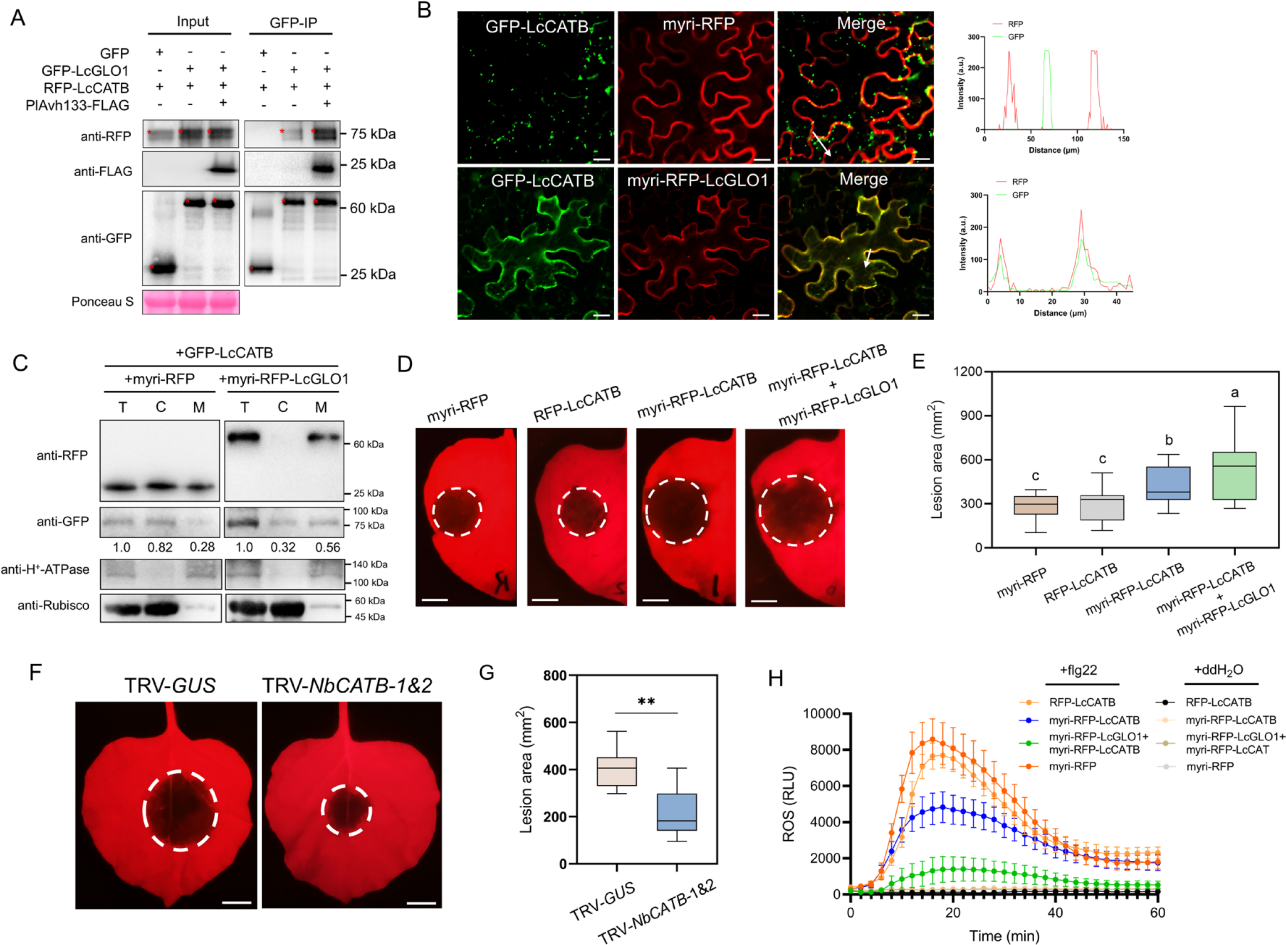
PM‐localised LcGLO1‐LcCATB complex promotes plant susceptibility by eliminating H_2_O_2_. (A) In vivo co‐IP of LcGLO1 with LcCATB. Total proteins were extracted from *N. benthamiana* leaves expressing GFP‐LcGLO1 or GFP (control) together with RFP‐LcCATB. The immune complexes were pulled down using anti‐GFP agarose beads. Protein loading is indicated by Ponceau S staining. Expected protein bands are indicated by red asterisks. (B) PM‐localised LcGLO1 recruits LcCATB to the PM. Cells of *A. tumefasciens* harbouring GFP‐LcCATB were co‐infiltrated with cells of *A. tumefasciens* harbouring myri‐RFP‐LcGLO1 or myri‐RFP into *N. benthamiana* leaves. Photographs were taken at 48 hpa. Scale bars, 20 μm. The fluorescence intensity charts on the right correspond respectively with the white arrow cross‐sections in the images to their left. (C) Subcellular fractionation of extracts from *N. benthamiana* leaves co‐expressing LcCATB with PM‐localised LcGLO1 or myri‐RFP. C, cytosolic fraction; M, microsomal fraction; T, total protein; (D, E) PM‐localised LcCATB promoted plant susceptibility to *P. capsici*. *N. benthamiana* leaves expressing myri‐RFP‐LcCATB, RFP‐LcCATB, and myri‐RFP separately, or leaves co‐expressing myri‐RFP‐LcCATB with myri‐RFP‐LcGLO1, were inoculated with *P. capsici*. Lesion development was measured and leaves photographed at 48 hpi. Scale bar, 1 cm. Different letters indicate significant differences using Duncan's multiple range test at *p* < 0.01; *n* ≥ 10. (F, G) Silencing of *NbCATB‐1* and *NbCATB‐2* enhances resistance to *P. capsici*. The lesions in *N. benthamiana* leaves were measured and photographed at 48 hpi. Scale bar, 1 cm. Asterisks indicate significant differences (**p < 0.01; Student's *t*‐test; *n* ≥ 12). (H) ROS burst induced by flg22 in *N. benthamiana* leaves co‐expressing myri‐RFP‐LcCATB with myri‐RFP‐LcGLO1, or leaves expressing RFP‐LcCATB, myri‐RFP‐LcCATB and myri‐RFP separately. Relative luminescence units (RLU) indicate relative amounts of H_2_O_2_ production induced by 10 μM flg22 or ddH_2_O in leaf strips. The results shown are representative of three independent experiments. Each data point consists of 8 replicates. Error bars indicate SD. In E and G, the central horizontal line denotes the median; vertical box height corresponds to the interquartile range; and the whiskers show the maximum and minimum values within the analysed dataset.

It has been demonstrated that GLOs can interact with CATs in rice to rapidly decompose H_2_O_2_ (Zhang et al. 2016; Li et al. 2022). Therefore, we hypothesised that PM‐localised LcGLO1 would interact with litchi CAT(s) in the PM for compromising plant immunity. To verify this hypothesis, we searched for litchi homologous CAT(s) of rice CATB and CATC using the Sapinaceae Genomic DataBase (http://www.sapindaceae.com) and identified one CAT that showed high similarity, which we named LcCATB (gene ID: LITCHI009991; Figure S9). We then investigated whether LcGLO1 interacts with LcCATB through co‐IP. The results showed that LcCATB co‐precipitated with LcGLO1 (Figure 5A). We also found that PlAvh133, LcGLO1 and LcCATB were able to form a complex in vivo and the association of LcGLO1‐LcCATB was enhanced by PlAvh133 (Figure 5A). To test whether PlAvh133 directly interacts with LcCATB, GST pull‐down was performed to detect complex formation between His‐PlAvh133 and GST‐LcCATB proteins. However, His‐PlAvh133 could not interact with GST‐LcCATB in vitro (Figure S10A). Subsequently, we transiently expressed GFP‐LcCATB in *N. benthamiana* leaves in the presence or absence of myri‐RFP‐LcGLO1, and the confocal observations confirmed that GFP‐LcCATB relocated to the PM only when co‐expressed with myri‐RFP‐LcGLO1 (Figure 5B; Figure S10B,C). Subcellular fractionation also showed that more GFP‐LcCATB was detected in microsomes in the presence of myri‐RFP‐LcGLO1 (Figure 5C).

To test whether altered subcellular localisation of LcCATB would affect plant resistance, the N‐terminal RFP‐LcCATB was fused to the myri sequence. By confocal microscopy and subcellular fractionation, we observed that the fusion protein myri‐RFP‐LcCATB was localised to the PM (Figure S10D,E). Subsequently, we transiently expressed myri‐RFP‐LcCATB, RFP‐LcCATB, and the control myri‐RFP in the *N. benthamiana* leaves and performed the infection assays. The results showed that the lesions in myri‐RFP‐LcCATB‐expressing leaves were significantly larger than those in the myri‐RFP‐expressing leaves (Figure 5D,E; Figure S10F). However, there were no significant differences in lesion area between the RFP‐LcCATB‐expressing leaves and the control (Figure 5D,E; Figure S10F). More importantly, leaves co‐expressing myri‐RFP‐LcCATB and myri‐RFP‐LcGLO1 exhibited the most susceptibility to *P. capsici* (Figure 5D,E; Figure S10F). We searched for the *N. benthamiana* homologue of *LcCATB*, and identified two *NbCATB*, *NbCATB‐1* (Gene ID: Niben101Scf01497g03008.1) and *NbCATB‐2* (Gene ID: Niben101Scf02041g04015.1), that showed high similarity to *LcCATB* (Figure S11). Due to the high sequence similarity between *NbCATB‐1* and *NbCATB‐2*, they could not be silenced individually. A construct, TRV‐*NbCATB*, was designed to silence both *NbCATB‐1* and *NbCATB‐2* via VIGS. qRT‐PCR analysis showed that *NbCATB‐1 and 2* were efficiently co‐silenced by the construct (Figure S12), and no obvious developmental phenotype was observed compared to the control. The infection assays revealed that the lesion areas in *NbCATB*‐silenced plants were smaller than those in the GUS control plants, which implied that knockdown of *NbCATB* promoted plant disease resistance (Figure 5F,G).

To evaluate whether the PM‐localised LcGLO1‐LcCATB complex facilitated pathogen infection by eliminating the ROS burst, we transiently expressed RFP‐LcCATB and myri‐RFP‐LcCATB independently and co‐expressed myri‐RFP‐LcCATB with myri‐RFP‐LcGLO1 in *N. benthamiana* leaves. Consistent with our observation in the infection assays, leaves co‐expressing myri‐RFP‐LcCATB with myri‐RFP‐LcGLO1 exhibited the lowest levels of ROS after flg22 treatment (Figure 5H). Leaves expressing myri‐RFP‐LcCATB also alleviated ROS accumulation but to a lesser extent (Figure 5H). Nevertheless, only a slightly reduced flg22‐triggered ROS burst was observed in the leaves expressing RFP‐LcCATB relative to the control (Figure 5H). These results support our hypothesis that the PM‐localised LcGLO1‐LcCATB complex accelerates the breakdown of ROS, thereby negatively regulating plant disease resistance.

### 2.7 PM‐Localised LcGLO1 Destabilises LcRBOHD by Interacting With LcCPK5

**FIGURE 6:**
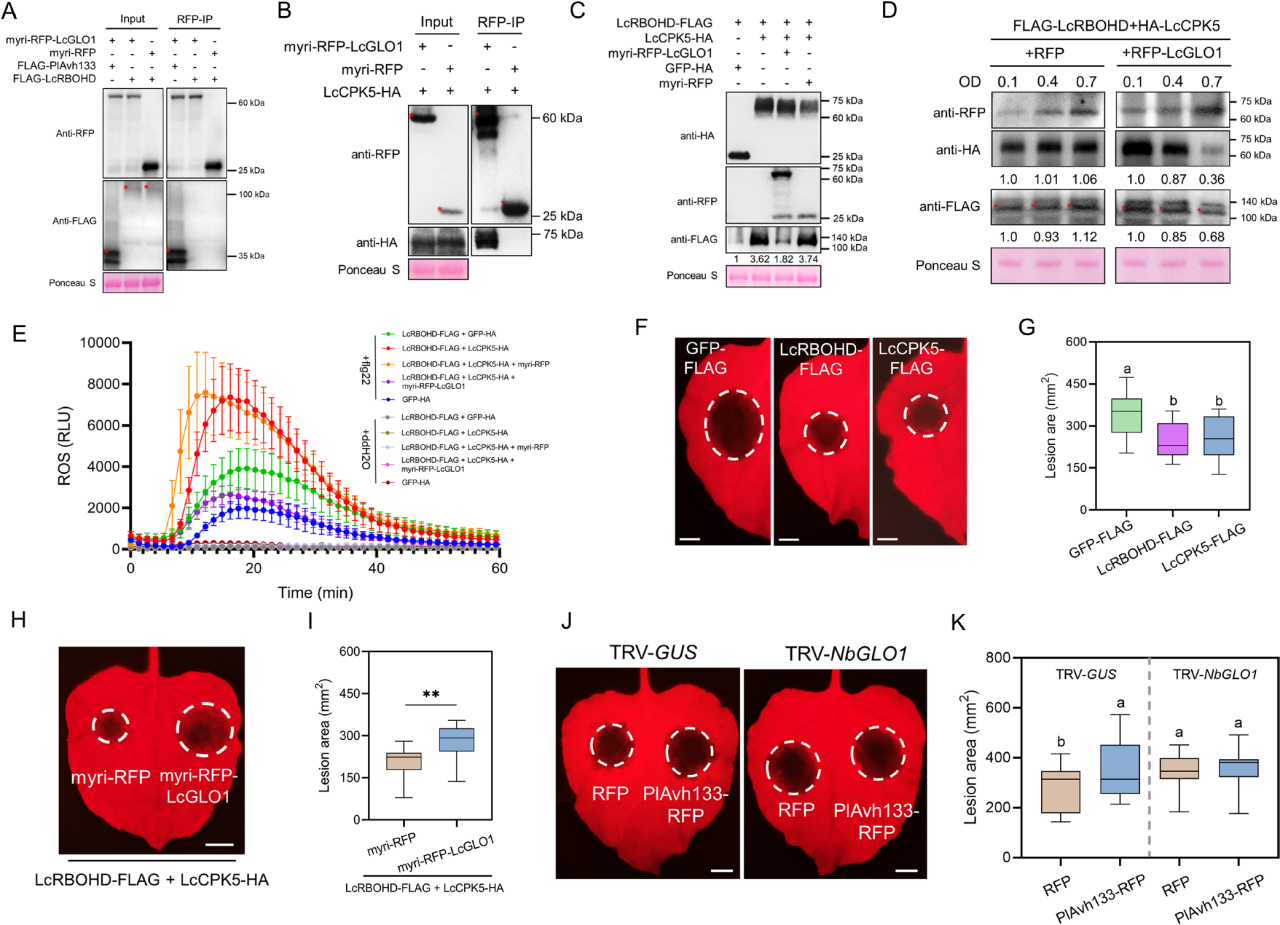
PM‐localised LcGLO1 destabilises LcRBOHD through binding with LcCPK5. (A, B) In vivo co‐IP of PM‐localised LcGLO1 with LcRBOHD or LcCPK5. Total proteins were extracted from *N. benthamiana* leaves expressing myri‐RFP‐LcGLO1 or myri‐RFP (control) together with LcRBOHD‐FLAG or LcCPK5‐FLAG. The immune complexes were pulled down using anti‐RFP agarose beads. Protein loading is indicated by Ponceau S staining. Expected protein bands are indicated by red asterisks. (C) PM‐localised LcGLO1 destabilises LcRBOHD by interacting with LcCPK5. Total proteins were extracted from *N. benthamiana* leaves expressing LcRBOHD‐FLAG + GFP, LcRBOHD‐FLAG + LcCPK5‐GFP, and LcRBOHD‐FLAG + LcCPK5‐GFP + myri‐RFP‐LcGLO1. The immune complexes were pulled down using anti‐GFP agarose beads. Protein loading is indicated by Ponceau S staining. The number represents the relative intensity. (D) LcGLO1 suppresses the protein accumulation of LcRBOHD by reducing the stability of LcCPK5. Total proteins were extracted from *N. benthamiana* leaves expressing LcRBOHD‐FLAG + LcCPK5‐HA + RFP and LcRBOHD‐FLAG + myri‐RFP‐LcGLO1 + LcCPK5‐HA. Protein loading is indicated by Ponceau S staining. The number represents the relative intensity. (E) ROS burst induced by flg22 in *N. benthamiana* leaves expressing LcRBOHD‐FLAG + GFP, LcRBOHD‐FLAG + LcCPK5‐GFP, and LcRBOHD‐FLAG + LcCPK5‐GFP + myri‐RFP‐LcGLO1. Relative luminescence units (RLU) indicate relative amounts of H_2_O_2_ production induced by 10 μM flg22 or ddH_2_O in leaf strips. The results shown are representative of three independent experiments. Each data point consists of 6 replicates. Error bars indicate SD. (F and G) Expression of LcRBOHD or LcCPK5 in *N. benthamiana* promotes resistance to *P. capsici*. *N. benthamiana* leaves expressing GFP‐FLAG, LcRBOHD‐FLAG or LcCPK5 were inoculated with *P. capsici*. Different letters indicate significant differences using Duncan's multiple range test at *p* < 0.01; *n* = 13. (H and I) Expression of PM‐localised LcGLO1 in *N. benthamiana* inhibits disease resistance caused by co‐expressing LcRBOHD and LcCPK5. *N. benthamiana* leaves expressing LcRBOHD‐FLAG + LcCPK5‐HA + myri‐RFP‐LcGLO1 or LcRBOHD‐FLAG + LcCPK5‐HA + myri‐RFP were inoculated with *P. capsici*. Asterisks indicate significant differences. **p < 0.01; Student's *t*‐test; *n* ≥ 12. (J, K) The virulence function of PlAvh133 depends on the plant GLO1. *P. capsici* was inoculated in the TRV‐*GUS*‐ or TRV‐*NbGLO1*‐silenced plants expressing PlAvh133‐RFP or RFP. Different letters indicate significant differences using Duncan's multiple range test at *p* < 0.01; *n* ≥ 13. In E, G and I, lesion development was measured and photographed at 48 hpi. Scale bar, 1 cm. In F, H and J, the central horizontal line denotes the median; vertical box height corresponds to the interquartile range; and the whiskers show the maximum and minimum values within the analysed dataset.

Given that PM‐localised LcGLO1 is sufficient to suppress ROS burst and the ROS‐scavenging ability of plants co‐expressing PM‐localised LcGLO1 and LcCATB was more than that of plants expressing PM‐localised LcCATB, we speculated that PM‐localised LcGLO1 has an additional route that interferes with the generation of ROS. RBOHD, a well‐known PM‐localised NADPH oxidase, catalyses the reduction of oxygen to superoxide, which in turn is converted to H_2_O_2_ by superoxide dismutase in the apoplast (Wu et al. 2023). To assess the ability of PM‐localised LcGLO1 to associate with LcRBOHD, we transiently co‐expressed myri‐RFP‐LcGLO1 and FLAG‐tagged LcRBOHD in planta followed by the co‐IP assays. Unexpectedly, myri‐RFP‐LcGLO1 could not be co‐purified with LcRBOHD‐FLAG (Figure 6A). Similarly, no interaction was observed between RFP‐PlAvh133 and LcRBOHD‐FLAG (Figure S13A). CPK5, a calcium‐dependent protein kinase has been documented to interact with and phosphorylate RBOHD, which is pivotal for the modulation of ROS production in *Arabidopsis* (Dubiella et al. 2013). Therefore, we tested the association between PM‐localised LcGLO1 and LcCPK5. The results of co‐IP showed that myri‐RFP‐LcGLO1, but not control myri‐RFP interacted with LcCPK5‐HA (Figure 6B). We further investigated whether PM‐localised LcGLO1 destabilises LcRBOHD by interacting with LcCPK5. The results showed that co‐expression with LcCPK5, but not GFP control, increased the protein levels of LcRBOHD after flg22 treatment (Figure 6C), confirming that LcCPK5 stabilises LcRBOHD in planta. However, the addition of PM‐localised LcGLO1 prevented the typical increase in RBOHD abundance (Figure 6C), indicating that PM‐localised LcGLO1 does inhibit the stabilisation of LcCPK5 toward LcRBOHD. To elucidate how LcGLO1 destabilises LcRBOHD, we co‐expressed LcCPK5 and LcRBOHD with myri‐RFP‐LcGLO1 in a dose‐dependent manner. The results showed that, compared to the RFP control, the protein levels of LcCPK5 decreased as the abundance of membrane‐localised LcGLO1 increased, accompanied by a concurrent reduction in LcRBOHD, relative to the RFP control. These results demonstrate that myri‐RFP‐LcGLO1 reduces LcRBOHD accumulation by promoting LcCPK5 degradation (Figure 6D). Furthermore, we found LcCPK5 positively regulated rapid, LcRBOHD‐mediated ROS production after flg22 stimulation (Figure 6E). As expected, there was reduced ROS accumulation after the addition of PM‐localised LcGLO1 (Figure 6E). Finally, to test the disease resistance function of LcRBOHD and LcCPK5, they were transiently expressed in *N. benthamiana* leaves, and infection assays were performed. The results demonstrated that the expression of LcRBOHD and LcCPK5 promotes plant disease resistance (Figure 6F,G; Figure S13B). However, the expression of PM‐localised LcGLO1 reversed the disease resistance caused by the co‐expression of LcRBOHD and LcCPK5 (Figure 6H,I; Figure S13C). Taken together, these data indicate a negative effect of PM‐localised LcGLO1 on RBOHD‐mediated ROS production through binding with LcCPK5.

To determine whether the ability of PlAvh133 to promote pathogen colonisation depends on GLO1, we transiently expressed PlAvh133‐RFP or RFP in the *NbGLO1*‐silenced or GUS control plants, and the leaves were harvested for infection assays. Unsurprisingly, the lesions in PlAvh133‐RFP‐expressing leaves were significantly larger than those in the RFP‐expressing leaves in the GUS control plants (Figure 6J,K; Figure S13D). However, there were no significant differences in lesion areas between them in the *NbGLO1*‐silenced plants (Figure 6J,K; Figure S13D), which indicated the virulence function of PlAvh133 depends on plant GLO1.

### 2.8 Transgenic Plants Overexpressing LcGLO1 Show Enhanced Plant Disease Resistance

**FIGURE 7:**
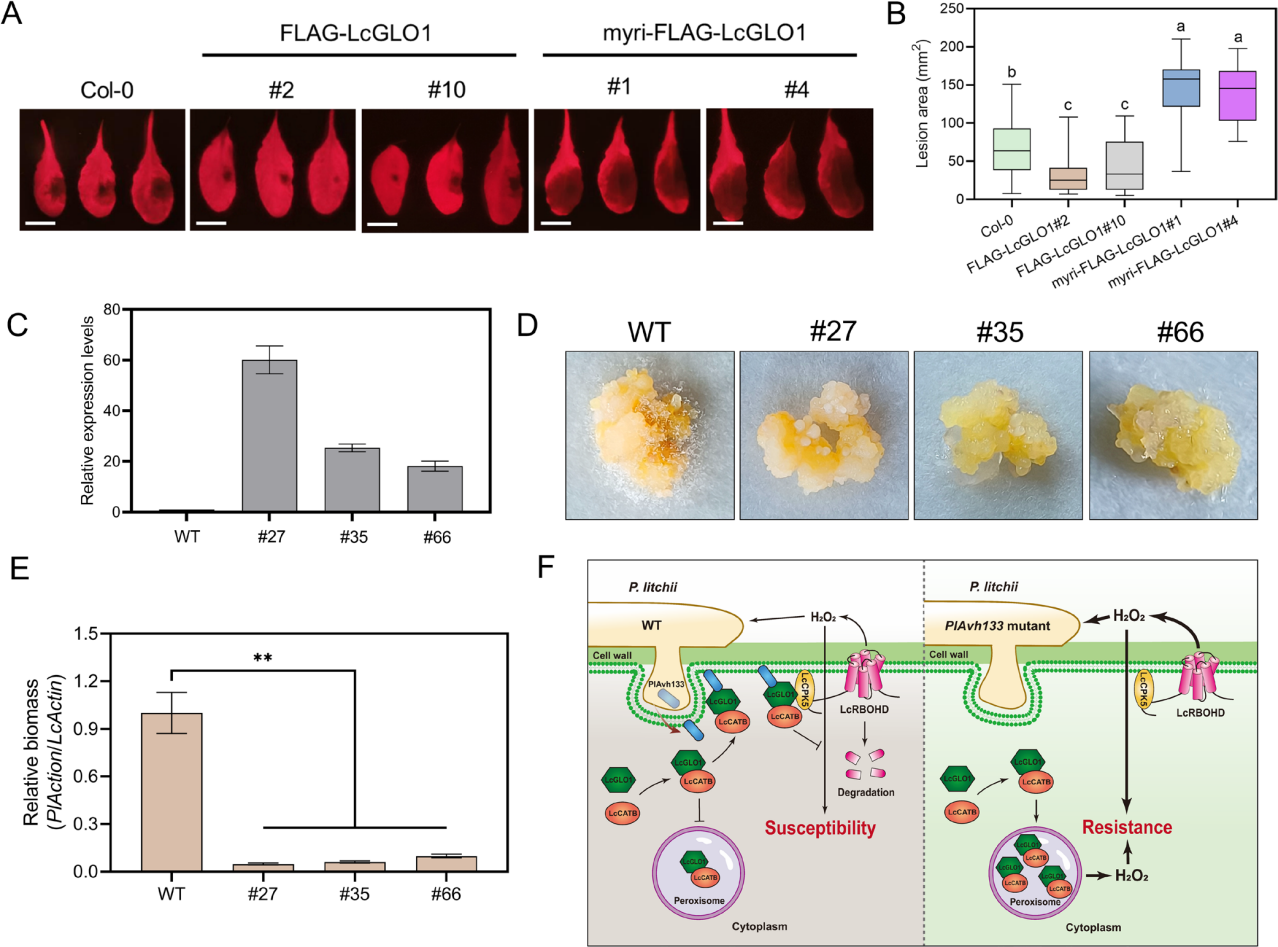
Overexpression of LcGLO1 in plants facilitates disease resistance. (A, B) The LcGLO1 transgenic Arabidopsis plants were more resistant to *P. capsici*. Four‐week‐old Arabidopsis seedlings were inoculated with *P. capsici* and lesion development was measured and photographed at 48 hpi. Scale bar, 1 cm. The central horizontal line denotes the median; vertical box height corresponds to the interquartile range; and the whiskers show the maximum and minimum values within the analysed dataset. Different letters indicate significant differences using Duncan's multiple range test at *p* < 0.01; *n* ≥ 19. (C) Relative transcript levels of *LcGLO1* were determined by qRT‐PCR in *LcGLO1* transgenic litchi calli. The relative expression levels were calibrated to the WT set as 1. The constitutively expressed gene, *LcActin*, was used as an internal reference. (D, E) The *LcGLO1* transgenic litchi calli were more resistant to *P. litchii*. Litchi calli were inoculated with zoospore suspensions from *P. litchii* WT strain SHS3. Disease symptoms were photographed at 72 hpi, and the relative biomass of *P. litchii* was determined by qPCR. Data represent means ± SD. Experiments were repeated three times with similar results. Asterisks indicate significant differences. **p < 0.01; Student's *t*‐test. (F) A proposed model for PlAvh133‐mediated plant immune suppression. In the absence of PlAvh133, LcGLO1 binds to LcCATB and co‐localises to the peroxisome, thereby participating in photorespiration and ROS burst. Upon infection, (1) effector PlAvh133 suppressed the enzymatic activity of LcGLO, and relocates LcGLO1 from peroxisome to the plasma membrane (PM), thereby reducing ROS burst in the peroxisome. (2) PM‐localised LcGLO1 recruits LcCATB to the PM to eliminate the ROS burst. (3) Additionally, PM‐localised LcGLO1 destabilises LcRBOHD through binding with LcCPK5, thereby further inhibiting ROS production and leading to the eventual enhancement of pathogenic colonisation. Solid arrows represent promotion; solid terminated lines represent inhibition.

To further characterise the function of LcGLO1 in planta, we introduced the RFP‐LcGLO1 and myri‐RFP‐LcGLO1 expression constructs into WT Arabidopsis (Col‐0) using *Agrobacterium*‐mediated transformation and generated Arabidopsis transgenic lines with RFP‐LcGLO1 and myri‐RFP‐LcGLO1 expression driven by the 35S promoter individually (Figure S14A,B). Four independent transgenic lines (FLAG‐LcGLO1 [#2 and #10] and myri‐FLAG‐LcGLO1 [#1 and #4]) were obtained, all of which showed normal growth. The LcGLO1‐expressing Arabidopsis showed more resistance to the *P. capsici* than WT plants (Figure 7A,B). Conversely, expression of PM‐localised LcGLO1 was more susceptible to the pathogen than the WT plants (Figure 7A,B). To further investigate the role of LcGLO1 in regulating litchi resistance to *P. litchii*, we generated LcGLO1 transgenic litchi overexpression lines (Figure 7C). Three independent transformant lines (#27, #35, #66) were obtained, all of which showed normal calli growth. Despite the *Agrobacterium*‐mediated transformation system for litchi having been established, plant regeneration remains challenging and time‐consuming. Therefore, the transformed litchi calli were used to conduct the infection assays directly. Transformed and non‐transformed litchi calli were inoculated with *P. litchii*, and their biomass was assessed at 72 hpi. The resistance of LcGLO1‐overexpression calli to *P. litchii* was significantly enhanced, evidenced by a marked reduction in the pathogen's biomass. (Figure 7D,E). Additionally, as expected, biomass accumulation of the PlAvh133 knockout mutants during litchi calli infection was significantly reduced compared to that of the wild‐type SHS3 (Figure S14C,D). Together, these results indicated that the expression of LcGLO1 is capable of facilitating plant defence against oomycete pathogens.

## 3 Discussion

Plants and pathogens have co‐evolved complex defence and counterdefence mechanisms throughout their interaction. Effectors secreted by pathogens manipulate plant defences by targeting different cell components. It is more challenging to conduct genetic manipulation and analyse gene functions in woody plants because of their long cycles and high difficulty. Hence, research on litchi resistance has long been lagging behind non‐woody plants. In this research, we assumed that the effector‐directed strategy can be used to identify novel regulatory components of plant immunity by characterising pathogenicity‐essential effectors and identifying their targets. We found that PlAvh133 was a virulence factor that promotes infection by *P. litchii*. Therefore, we screened for the host target protein using PlAvh133 as a probe. It has been widely accepted that ROS play vital roles in plant immune responses, which may be manipulated by pathogens to facilitate colonisation. For instance, two *P. sojae* CRN effectors, PsCRN63 and PsCRN115, interact with CATs and regulate H_2_O_2_ homeostasis in opposite directions (Zhang et al. 2015). *P. capsici* RxLR207 targets and promotes the degradation of BPA1‐Like proteins (binding partner of ACD11) in *A. thaliana*, which leads to the instability of ACD11 and disrupted ROS homeostasis (Li et al. 2019). Another effector, PpE18, secreted by *P. parasitica*, directly inhibited the ROS‐scavenging activity of a peroxisome membrane‐associated ascorbate peroxidase and destroyed ROS levels in the peroxisome (Cao, Zhang, et al. 2024). The *P. infestans* RxLR effector Pi16275 reduces ROS accumulation by decreasing the expression of the potato ribosomal protein StRPS5 (Liu et al. 2025). Our study provides another molecular framework adopted by *P. litchii* where the RXLR effector PlAvh133 targets LcGLO1 to suppress the ROS burst and plant immunity by three distinct molecular strategies. The proposed model involves the relocation of LcGLO1 to the PM and its enzyme activity suppression. In the PM, LcGLO1 eliminates ROS by binding with LcCATB on the one hand, and inhibits the LcCPK5‐mediated LcRBOHD protein accumulation on the other hand, thereby disturbing the generation of ROS (Figure 7F).

Transient expression of LcGLO1 in *N.benthamiana* enhanced the flg22‐induced ROS burst, yet only marginally reduced lesion size and biomass during oomycete infection (no significant difference). In contrast, stable transgenic Arabidopsis lines and litchi calli over‐expressing LcGLO1 exhibited a clear and significant increase in resistance. These data collectively support LcGLO1 as a positive regulator of plant immunity. The discrepancy observed in the infection assay results between the transient and stable systems might be attributed to the higher and more stable LcGLO1 accumulation in transgenic plants than in transient assays, or species‐specific differences in the downstream immune components that execute GLO1‐mediated defence between litchi and *N. benthamiana*. The observed discrepancy between the transient and stable overexpression assays prompts the recommendation that subsequent functional studies of immunity‐related genes prioritise the use of stably transformed litchi lines.

Plant PM are at the forefront of the plant‐pathogen battlefield. In the early stages of infection, a large amount of H_2_O_2_ is produced from membrane‐associated respiratory burst oxidase homologues (RBOHs) which are the functional equivalents of mammalian NADPH oxidases (Yasuhiro et al. 2015). Although H_2_O_2_ is primarily produced in the apoplast due to the activation of RBOHs, recent analyses showed that H_2_O_2_ is the mobile signal that sulfenylates transcription factor CHE in a concentration‐dependent manner (Cao, Karapetyan, et al. 2024). This modification serves as a molecular switch that activates CHE‐mediated SA increase and subsequent Pip accumulation in systemic tissues to synergistically induce systemic acquired resistance (SAR). Another study found that rice ROD1, a Ca^2+^ sensor protein, suppresses rice immunity via promoting localisation of CatB to the PM and stimulating catalase activity to eliminate ROS (Gao et al. 2021). The fungal effector AvrPiz‐t structurally mimics ROD1 and activates the same ROS‐scavenging cascade to suppress host immunity and promote virulence (Gao et al. 2021). In our study, the recruitment of GLO‐CAT complex to the PM would also constitute a shortcut that enables spatial precision in H_2_O_2_ degradation, and the inhibition of immune signaling. The suppression of flg22‐mediated ROS burst by the PM‐localised LcGLO1 and LcCATB provides strong evidence for the above speculation. On the other hand, our results demonstrate that there is a crosstalk between the photorespiratory pathway component and RBOHD when they are in the PM. Through binding with LcCPK5, LcGLO1 suppresses the LcCPK5‐mediated LcRBOHD protein accumulation, resulting in reduced H_2_O_2_ production. This indicates that when pathogen effectors bind to host target proteins, they could suppress host immune responses via multiple mechanisms. In *Arabidopsis thaliana* , CPK5 involvement in RBOHD S39 and S148 phosphorylation is induced by externally applied H_2_O_2_ (Dubiella et al. 2013). We speculate that the binding of PM‐localised LcGLO1 to LcCPK5 inhibits LcCPK5‐mediated phosphorylation of LcRBOHD, thereby rendering LcRBOHD more susceptible to degradation.

Although PM‐localised LcCATB is able to eliminate ROS, PM‐localised LcGLO1 may also produce a large amount of ROS. Inhibition or use of host protein enzyme activity is demonstrated to be one of the pathogen effector modes of action (Li, Wang, et al. 2023; Xiao et al. 2024). We therefore tested and demonstrated that PlAvh133 directly inhibits the enzymatic activity of LcGLO1 by approximately 40%. Similar results were also obtained in the studies of *Barley Stripe Mosaic Virus γb* protein (Yang, Li, et al. 2018). Moreover, the association of LcGLO1‐LcCATB synergised by PlAvh133 might promote reaction rates to eliminate H_2_O_2_. Previous studies reveal that the association‐dissociation equilibrium between GLO and CAT serves as a key switch for modulating intracellular H_2_O_2_ levels (Zhang et al. 2016). Specifically, dissociation of GLO from CAT elevates H_2_O_2_ production. In our study, PlAvh133 enhances the physical binding between LcGLO1 and LcCATB. We therefore propose that this proximity enables LcCATB to more efficiently scavenge H_2_O_2_ directly at the site of its generation by LcGLO1, thereby reducing local ROS bursts. According to the substrate channelling mechanism, once enzyme–enzyme complexes dissociate, channelling is disrupted and intermediate (H_2_O_2_) will quickly equilibrate with the bulk medium resulting in increased concentrations of the intermediate (H_2_O_2_) in the bulk solution (Zhang 2011). Conversely, a stable complex association facilitates more efficient H_2_O_2_ elimination via substrate channelling. This spatial proximity of enzyme complexes likely explains the enhanced H_2_O_2_ elimination efficiency. Structural and biochemical analysis may elucidate the molecular basis of how these effectors affect the enzymatic activity of GLO‐CAT complex. In future work, gene editing could be employed to construct mutants of plant GLO that cannot be targeted by the effectors but retain full enzymatic activity. These engineered plants may possess disease resistance and significant application potential in plant production.

Functioning independently from RBOHs, the GLO‐CAT complex was demonstrated to act as a switch to modulate H_2_O_2_ levels in plants (Sewelam et al. 2014; Zhang et al. 2016; Li et al. 2022). In comparison to GLO‐CAT dissociation, less H_2_O_2_ was produced when GLO was bound to CAT (Zhang et al. 2016). However, different plant isozymes of GLO show distinct physiological functions and characteristics. For example, GLO1 and GLO4, two important photorespiratory regulators in rice, harbour the highest enzymatic activities among rice GLOs, and both knockout mutants exhibited obvious dwarfism phenotypes (Zhang et al. 2017). Nevertheless, among GLO3 or GLO5 overexpression and RNAi lines, only GLO3 overexpression lines showed significantly increased L‐lactate‐oxidising activity but no other noticeable phenotypic changes (Zhang et al. 2017). The studies of GLOs in *N. benthamiana* and *A. thaliana* indicated that each of them could play different roles in the production of H_2_O_2_ and defence (Rojas et al. 2012; Xu et al. 2018). Although most of the evidence shows that GLOs contribute to disease resistance (Rojas et al. 2012; Ahammed et al. 2018; Yang, Wang, et al. 2018), silencing of *GLO1* in rice results in enhanced resistance to *Xanthomonas oryzae* pv oryzae (Jiang et al. 2023). Here, we demonstrated that LcGLO1 functions as a positive regulator of plant basal immunity, as evidenced by the enhanced disease resistance observed in both Arabidopsis and litchi calli overexpressing LcGLO1. On the one hand, overexpression of PM‐localised LcGLO1 enhanced plant disease susceptibility. Our BLAST analysis found that there were 7 GLO‐encoding genes in the litchi genome. Given that GLO isozymes could interact with each other or form protein complexes with other photorespiratory enzymes (Zhang et al. 2012), further exploration is also needed on whether other LcGLOs and photorespiratory enzymes can be classified as immunity regulators that confer broad‐spectrum resistance to various plant pathogens.

## 4 Conclusion

In summary, our results showed that the RXLR effector PlAvh133, a key virulence factor of *P. litchii*, could target the host GLO1 and inhibit its enzymatic activity. PlAvh133 recruits the LcGLO1‐LcCATB complex to the PM to eliminate the ROS burst. Additionally, PM‐localised LcGLO1 destabilises LcRBOHD through binding with LcCPK5, thereby further inhibiting ROS production and leading to the eventually enhanced pathogenic colonisation. The overexpression of LcGLO1 in litchi calli enhanced resistance to litchi downy blight. This finding not only advances our understanding of the pathogenic mechanisms of phytopathogenic oomycete but also reveals that the subcellular localisation of GLO1 could determine its roles in plant immunity. Future studies will continue to systematically evaluate the resistance of LcGLO1‐overexpressing litchi regenerated plantlets and their fruits against downy blight and other diseases, which will represent a milestone advancement in litchi disease‐resistant breeding.

## 5 Materials & Methods

### 5.1 Microbial Strains and Plant Growth Conditions

The *P. litchii* strain SHS3 and *P. capsici* strain LT263 were routinely grown on carrot juice agar (CJA) at 25°C in the dark. *Agrobacterium tumefaciens* strain GV3101 was cultured on Luria‐Bertani (LB) agar or broth supplemented with appropriate antibiotics and incubated at 28°C. *Escherichia coli* strains BL21 and JM109 were cultured on LB medium at 37°C, supplemented with appropriate antibiotics. *N. benthamiana* and *A. thaliana* Col‐0 plants were grown in a controlled climate chamber at 19°C–22°C with a photoperiod of 18 h light/6 h darkness for 4–6 weeks.

### 5.2 *Peronophythora Litchii* Transformation and Pathogenicity Tests

Mutants of *Peronophythora litchii* were generated using PEG‐mediated protoplast transformation as described previously (Jiang et al. 2017; Situ et al. 2023). The tender leaves of litchi (‘GuiWei’) were inoculated with zoospore suspensions (100 zoospores) of the wild type or mutants, and maintained at 25°C. Photographs were taken 2 days after inoculation and were collected for biomass evaluation.

### 5.3 RNA and DNA Extraction, and qRT‐PCR

Infected leaf samples or *Peronophythora litchii* cultures were ground in liquid nitrogen and used for RNA or DNA isolation. Genomic DNA was extracted using a genomic DNA kit following procedures described by the manufacturer (Omega Bio‐Tek). Total RNA was isolated using an All‐In‐One DNA/RNA Mini‐preps Kit (Bio Basic) and then used as templates for cDNA synthesis. Reverse transcription was performed using the PrimeScript RT reagent Kit (Takara). qRT‐PCR was performed on a qTOWER3 Real‐Time PCR thermal cycler (Analytik Jena) using ChamQ SYBR Colour qPCR Master Mix (Vazyme) under the following conditions: 95°C for 2 min; 40 cycles at 95°C for 30 s, 60°C for 30 s, followed by a dissociation program of 95°C for 15 s, 60°C for 1 min, and 95°C for 15 s to obtain melt curves. *PlActin*, *LcActin* or *NbEF1α* was used as the internal control. Data were analysed using the $2^{-\Delta \Delta C_{t}}$ method. The primers used in this study are listed in Table S2.

### 5.4 Agroinfiltration Assay in *N. Benthamiana*

The recombinant plasmid was introduced into *A. tumefaciens* strain GV3101 by heat shock. *A. tumefaciens* carrying the respective recombinant plasmids was cultured in LB broth at 28°C with shaking at 200 rpm for 48 h. The cultures were harvested and washed 3 times with infiltration buffer (10 mM MES, 10 mM MgCl_2_, and 200 mM acetosyringone), and then resuspended in infiltration buffer to achieve the final concentration OD_600_ = 0.5 for agroinfiltration into *N. benthamiana* leaves. After 2 days, the leaves were then inoculated with *P. capsici* as stated above, and incubated for another 2 days. For these infection assays, the infiltrated and inoculated *N. benthamiana* leaves were viewed and photographed under UV light to visualise fluorescence, which helped improve detection of infected areas, and then lesion area was measured manually at 48 h after inoculation. All samples were analysed using three independent biological replicates.

### 5.5 Co‐Immunoprecipitation and LC–MS/MS Analyses

For the co‐IP experiments, *N. benthamiana* leaves were infiltrated with constructs at a final OD_600_ of 0.5. The samples were frozen in liquid nitrogen 24 h after agroinfiltration. Proteins were extracted with lysis buffer [25 mM Tris‐HCl, pH 7.5, 150 mM NaCl, 1 mM ethylenediaminetetraacetic acid (EDTA), 0.2% NP‐40, 10% glycerol and protease inhibitor cocktail (Sigma‐Aldrich)] and centrifuged at 4°C for 15 min at 13 000 *g*. The sample supernatants were incubated with 20 μL anti‐GFP/RFP beads (GFP/RFP‐Trap_A beads; Chromotek) for 3 h with gentle agitation at 4°C. The beads were collected using DynaMag magnet and were washed five times with wash buffer [25 mM Tris‐HCl, pH 7.5, 150 mM NaCl, 1 mM EDTA, 10% glycerol and protease inhibitor cocktail (Sigma‐Aldrich)]. Bound protein complexes were eluted and boiled in 5× sodium dodecylsulfate loading buffer for 10 min. Proteins were analysed by Western blotting. For the LC–MS/MS analyses, the bound proteins were eluted with the addition of 50 μL glycine (0.2 M, pH = 2.5) for 40 s, and were neutralised by the addition of 5 μL Tris base (1 M, pH = 10.4). The resulting peptide mixtures were analysed by high‐performance liquid chromatography and ion‐trap mass spectrometry.

### 5.6 Luciferase Complementation Assay

The assay was performed as previously described (Li et al. 2014). *A. tumefaciens* strain GV3101 containing the indicated plasmids was infiltrated into expanded leaves of *N. benthamiana* and incubated in the growth room for 48 h before the LUC activity measurement. For the CCD imaging, 1 mM luciferin was sprayed onto the leaves. The in vivo Plant Imaging System LB985 (Berthold) was used to photograph the bioluminescence signals in the detached leaves. For LUC activity measurements, relative LUC activity per cm^2^ in infiltrated leaf area was calculated by the Infinite 200 PRO microplate reader (TECAN). Each data point contained at least four replicates, and three independent experiments were carried out.

### 5.7 GST Pull‐Down Assays

For in vitro pull‐down assays, GST‐LcGLO1, GST‐LcGLO1L and His‐PlAvh133 were expressed in *E. coli* strain BL21 (DE3). Total proteins of *E. coli* expressing GST‐LcGLO1 or GST‐LcGLO1L were extracted using lysis buffer (10 mM Tris pH 7.5, 150 mM NaCl, 1 mM protease inhibitor phenylmethylsulfonyl fluoride), and incubated with 20 mL of glutathione agarose beads (Invitrogen) at 4°C for 3 h. The supernatant was then removed and the beads were added to the supernatant of the total protein extract from *E. coli* expressing His‐PlAvh133 and incubated for 3 h at 4°C. Bound protein complexes were eluted and boiled in 5 × sodium dodecylsulfate loading buffer for 10 min. Proteins were analysed by Western blotting.

### 5.8 Fluorescence Detection by Confocal Microscopy

The subcellular localisation of PlAvh133, LcGLO1 and LcCATB, was analysed in *N. benthamiana* leaves and in *A. thaliana* protoplasts. For *N. benthamiana* leaves, *A. tumefaciens* ‐mediated infiltration was performed as described before. The final OD_600_ for each strain was 0.1 for confocal imaging. For *A. thaliana* protoplasts, isolation and polyethylene glycol‐mediated transfection were performed as described in Yoo et al. (2007). Fluorescence in *N. benthamiana* leaves or *A. thaliana* protoplasts was viewed with a Nikon A1 laser scanning microscope (Nikon). Red and green fluorescence were observed by excitation at 488 and 561 nm, respectively.

### 5.9 Flg22 Treatments and Oxidative Burst Assay

Leaves from 4‐week‐old *N. benthamiana* plants were sliced into 1 mm strips, incubated in 200 μL of water in a 96‐well plate under weak light for 12 h, and then 10 μM flg22 in 200 μL of luminescence detection buffer (100 mM luminol and 20 mg/mL horseradish peroxidase, Sigma‐Aldrich) was added. The equal volume of ddH_2_O was processed in the same manner and used as a mock control. Luminescence was recorded in the GLOMAX96 microplate luminometer (Promega) at 2‐minute intervals for 60 min.

### 5.10 VIGS In N. Benthamiana

We used the TRV‐based VIGS system, which involves bipartite sense RNA1 and RNA2 viruses, to silence target genes in *N. benthamiana*. The *NbGLO1* or *NbCATB* fragment was amplified by PCR and then cloned into the pTRV2 vector. At the 4‐leaf stage, the *N. benthamiana* plants were selected for agroinfiltration. Before agroinfiltration, *A. tumefaciens* cells carrying pTRV1 and pTRV2 constructs were collected, resuspended in infiltration buffer and mixed in a 1:1 ratio for agroinfiltration. Plants were grown for 2 weeks after infiltration under the same conditions stated above. The fully expanded leaves of the silenced plants were then used for inoculation (done as stated above) and qRT‐PCR assays. The TRV2‐*GUS* vector was used as the negative control.

### 5.11 GLO Enzymatic Activity Assay

Recombinant proteins His‐LcGLO1, His‐PlAvh133, His‐PlAvh202, and empty vector control used for enzymatic activity were produced in *E. coli* strain BL21 and purified using Ni NTA beads (Smart‐Lifesciences, Changzhou, China) according to the manufacturer's protocol. A 1.5 mL sample of the reaction mixture contained 66 mM phosphate buffer (pH 8.0), 1 mM 4‐amino‐antipyrine, 5 units of horseradish peroxidase, 2 mM phenol, 0.1 mM FMN, 5 mM glycolate/glyoxylate, and 0.05 mL of enzyme extract. The enzyme was added last to start the reaction and distilled water substituted for the substrate as the blank. H_2_O_2_ produced in the reaction mixture was determined spectrophotometrically at 520 nm and at 30°C.

### 5.12 *Agrobacterium tumefaciens* ‐Mediated Litchi Transformation

*Agrobacterium tumefaciens* ‐mediated litchi transformation was performed as described previously (Qin et al. 2025). The GV3101 strain of *Agrobacterium tumefaciens* containing the Ti plasmid was employed for plant transformation. Bacterial cultures harbouring either pCAMBIA1300‐eGFP or pCAMBIA1300‐eGFP‐LcGLO1 binary vectors were grown overnight at 28°C in YEP liquid medium (20 mL) supplemented with 50 mg/L kanamycin and 25 mg/L rifampicin, under continuous shaking (180 rpm). The cultures were harvested and subsequently resuspended in MS liquid medium containing 100 μM acetosyringone to achieve OD_600_ = 0.6. For transformation, embryonic calli underwent a 30‐minute immersion in the bacterial suspension before blot‐drying on sterile filter paper. Dark co‐cultivation proceeded for 2 days post‐infection. Selection was initiated by transferring treated embryos to antibiotic‐containing MS medium supplemented with 0.5 g/L activated charcoal, 10 mg/L hygromycin, and 300 mg/L timentin. Cultures were maintained under controlled conditions (25°C ± 2°C) with 16‐hour daily illumination.

## Author Contributions

G.K., Z.J., X.P., J.S., Z.Z., and Y.S. conceived the original idea and designed the experiments. J.S., Z.Z., Y.S., J.F., M.X., and F.Z. conducted the experiments. J.S., Z.Z., and Y.S. analysed data and wrote the paper. G.K., Z.J., X.Z., X.P., G.H., J.Z., M.L., P.X., W.L., and P.L. reviewed and edited the manuscript; all authors provided detailed comments.

## Conflicts of Interest

The authors declare no conflicts of interest.

## Supporting information


**Appendix S1:** pbi70457‐sup‐0001‐AppendixS1.doc.

## Data Availability

The data that support the findings of this study are available on request from the corresponding author. The data are not publicly available due to privacy or ethical restrictions.
