# Supplementary material for: Effector‐Mediated Spatial Reprogramming of Glycolate Oxidase Subverts Peroxisomal and Membrane‐Associated ROS Defences
Source: Plant Biotechnol J. 2025 Nov 12;24(4):1919–35. doi: 10.1111/pbi.70457 (PMC13140691; doi:10.1111/pbi.70457)
Supplement: Supplementary file 1 — Appendix S1: pbi70457‐sup‐0001‐AppendixS1.doc. [file PBI-24-1919-s001.doc]

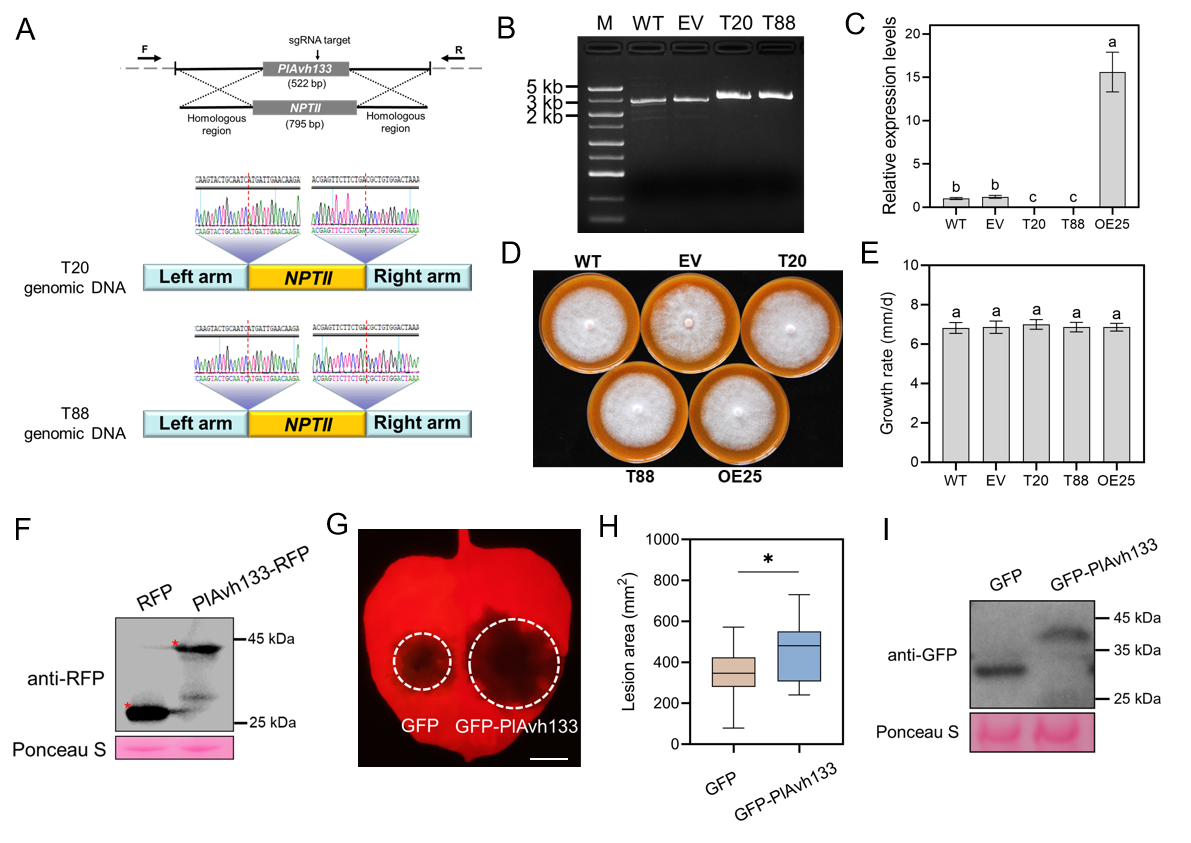


**Supplemental figure 1. Knockout of *PlAvh133* by CRISPR/Cas9.** (A and B) Schematic diagram of the gene replacement at the *PlAvh133* locus by CRISPR/Cas9, and PCR analysis of the *PlAvh133* knockout mutants. (C) Relative transcript levels of *PlAvh133* were determined by qRT-PCR in WT, EV, T20, T88 and OE25. The relative expression levels were calibrated to MY set as 1. The constitutively expressed gene, *PlActin*, was used as internal reference. Different letters indicate significant differences (p < 0.01; Duncan’s multiple range test). (D and E) The phenotypes of *PlAvh133* mutants were identical to WT. Photographs were taken at 5 day post-inoculation. Same letters at the top of the bars represent no significant differences (*p* > 0.05; Duncan’s multiple range test). (F) Immuno-detection of PlAvh133-RFP in *N. benthamiana* leaves. Anti-RFP was used to detect the expression of PlAvh133-RFP and RFP proteins. Protein loading is indicated by Ponceau S staining. Expected protein bands are indicated by red asterisks. Experiments were repeated three times with similar results. (G and H) Expression of PlAvh133 in *N. benthamiana* enhanced susceptibility to *P. capsici*. *N. benthamiana* leaves expressing GFP-PlAvh133 or GFP were inoculated with *P. capsici*. Lesion development was measured and photographed at 48 hpi. Scale bar, 1 cm. Asterisks indicate significant differences. *p<0.05; Student’s t-test; n=21. The central horizontal line denotes the median; vertical box height correspond to interquartile range, respectively; and the whiskers show the maximum and minimum values within the analyzed dataset. (I) Anti-GFP was used to detect the expression of GFP-PlAvh133 and GFP proteins in *N. benthamiana* leaves. Protein loading is indicated by Ponceau S staining. Experiments were repeated three times with similar results.


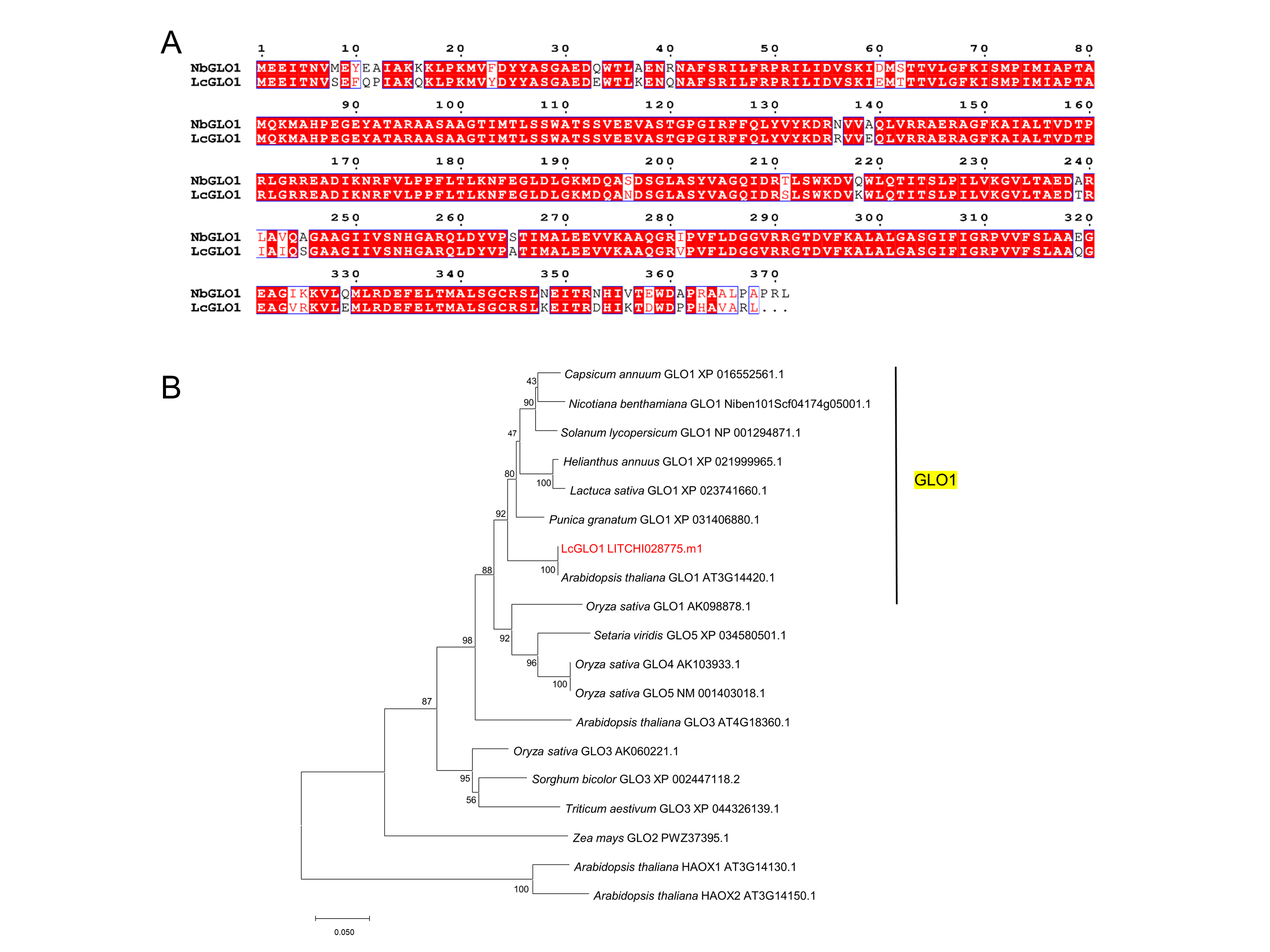


**Supplemental figure 2. Phylogenetic analysis of LcGLO1 and homologous proteins from other plants.** (A) Protein sequence alignment of NbGLO1 and LcGLO1. The sequence alignment was generated in Clustal W, and fully conserved sites are shown with red background, while sites with red font have similar amino acids. (B) A Neighbor-joining tree was constructed using MEGA X. Percent bootstrap values are shown at the branch points out of 1000 bootstrap replications. *A. thaliana* HAOX1&2 were used as outgroup. LcGLO1 from lychee is shown in red and GLO1 homologs are indicated with the vertical bar on the right. A genetic distance scale is shown on the bottom left.


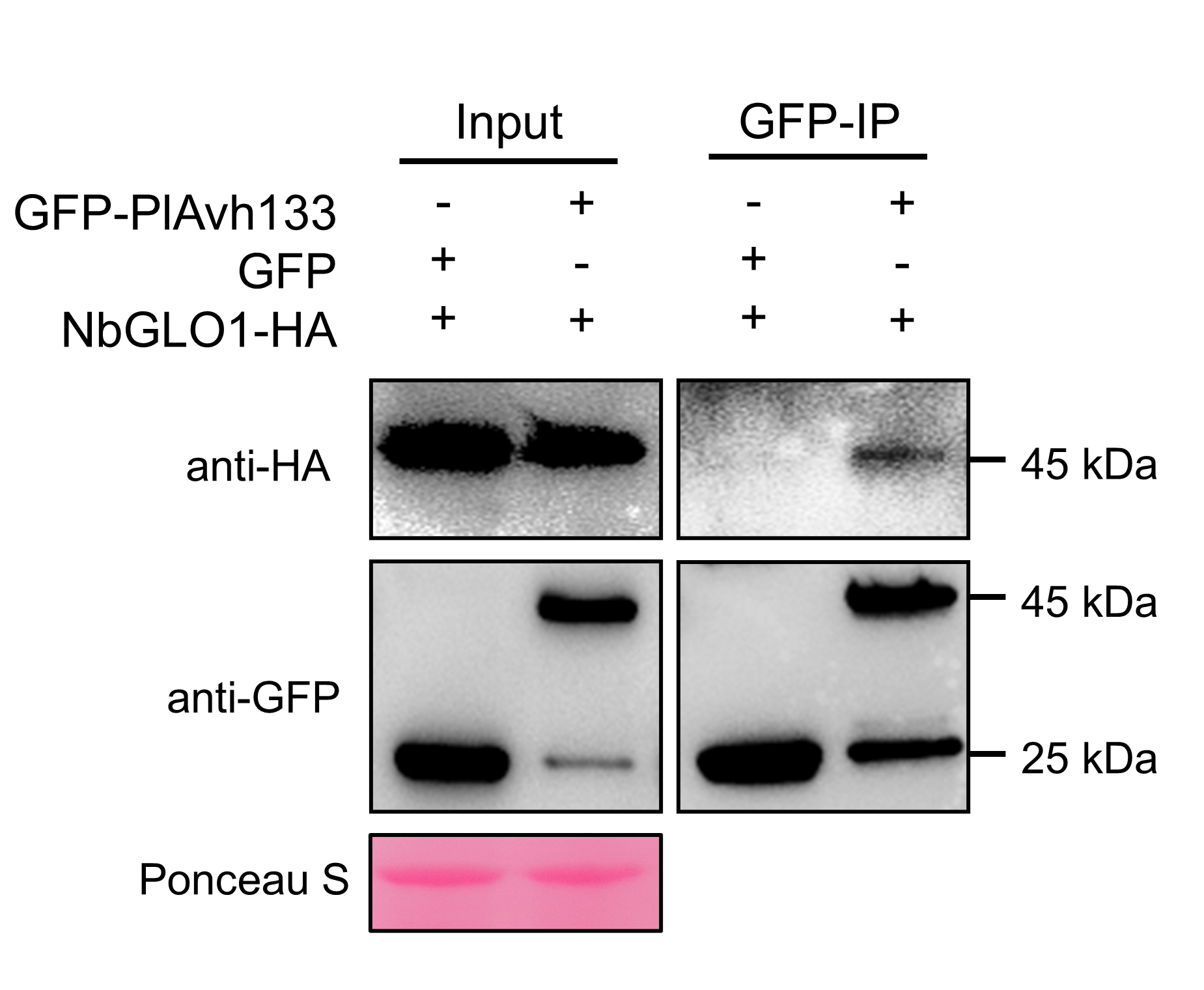


**Supplemental figure 3. *In vivo* co-IP of PlAvh133 with NbGLO1.** Total proteins were extracted from *N. benthamiana* leaves expressing GFP-PlAvh133 or GFP (control) together with NbGLO1-HA. The immune complexes were pulled down using anti-GFP agarose beads. Protein loading is indicated by Ponceau S staining.


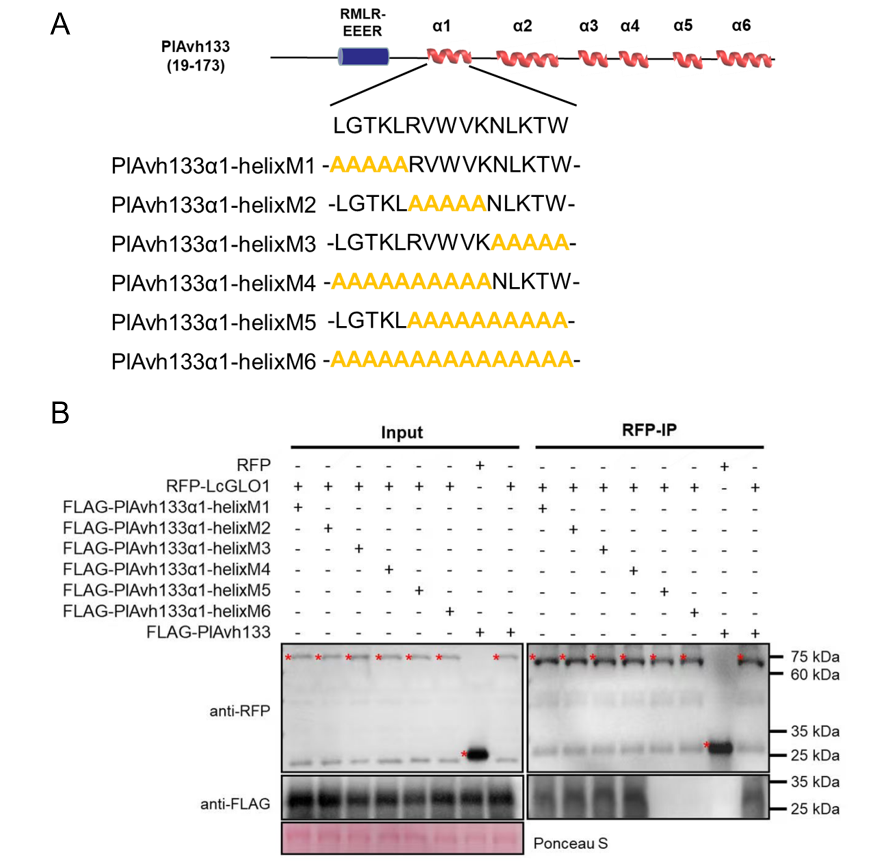


**Supplemental figure 4.** **Co-IP assay between PlAvh133 α1-helix mutants and LcGLO1.** (A) Schematic view of the PlAvh133 α1-helix mutants. (B) Co-IP assay between PlAvh133 α1-helix mutants and LcGLO1. Total proteins were extracted from *N. benthamiana* leaves expressing RFP-LcGLO1 or RFP (control) together with PlAvh133-FLAG or PlAvh133-FLAG derivatives. The immune complexes were pulled down using anti-RFP agarose beads. Protein loading is indicated by Ponceau S staining. Expected protein bands are indicated by red asterisks.


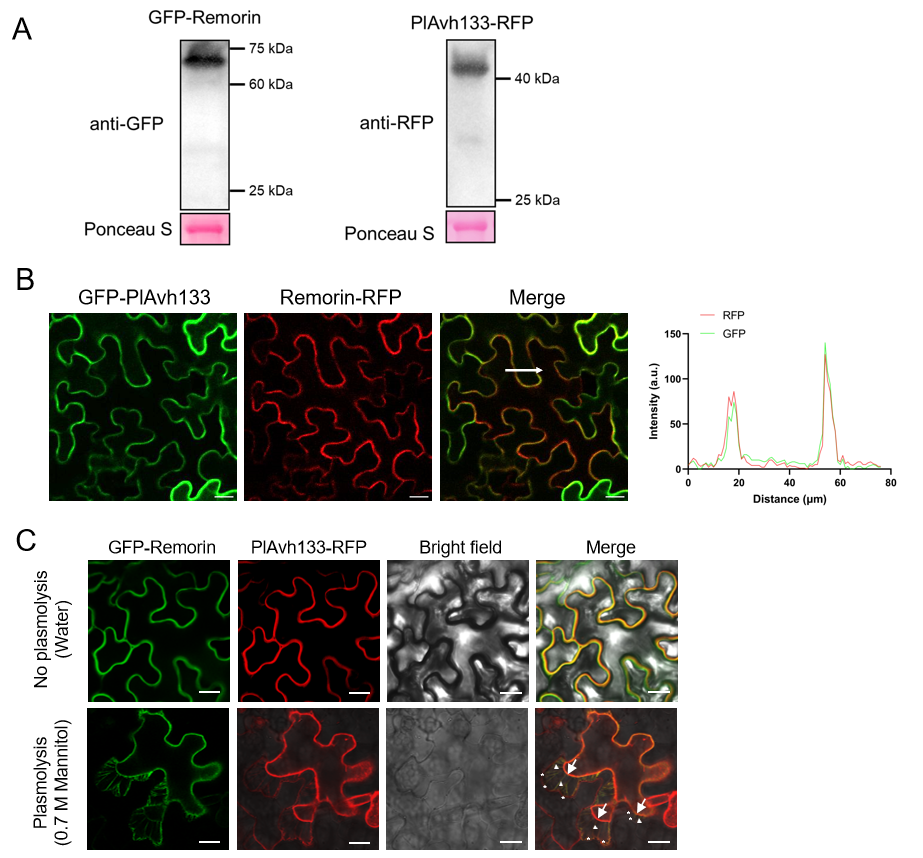


**Supplemental figure 5. Subcellular localization of PlAvh133 in *N. benthamiana* leaf cells.** (A) Anti-GFP and anti-RFP was used to detect the expression of GFP-Remorin and PlAvh133-RFP proteins, respectively. Protein loading is indicated by Ponceau S staining. Experiments were repeated three times with similar results. (B) Fluorescence in epidermal cells was detected by confocal microscopy 48 hpa. Scale bars, 20 μm. The fluorescence intensity charts on the right correspond respectively with the white arrow cross-sections in the images to their left. (C)PlAvh133-RFP and GFP- Remorin was subjected to confocal imaging after plasmolysis. *N. benthamiana* leaves were treated with 0.7 mol/L mannitol for plasmolysis. Fluorescent signals were observed at 36 hpa. Triangles indicate Hechtian strands, arrows indicate the PM region, and asterisks indicate the plant cell wall. Bars = 20 μm.


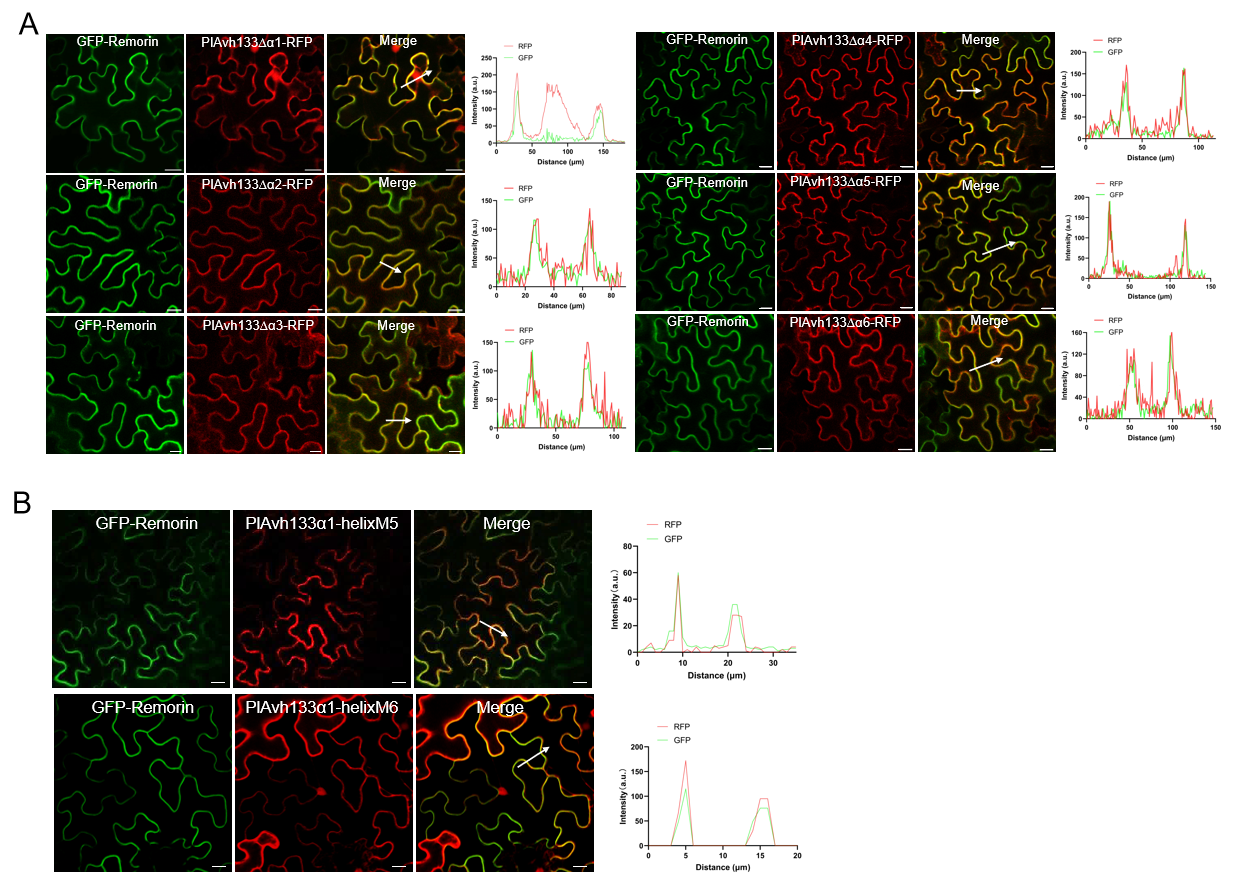


**Supplemental figure 6. Subcellular localization of PlAvh133 and its mutants.** (A and B) Fluorescence in epidermal cells was detected by confocal microscopy 48 hpa. Scale bars, 20 μm. The fluorescence intensity charts on the right correspond respectively with the white arrow cross-sections in the images to their left.


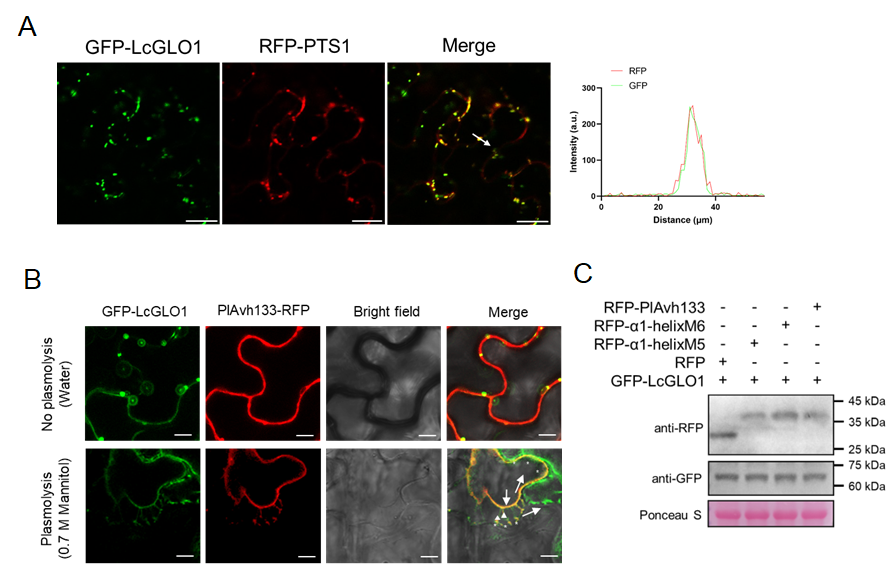


**Supplemental figure 7. Subcellular localization of LcGLO1 and immuno-detection of RFP-LcGLO1 in *N. benthamiana* leaves.** (A) Confocal images of *N. benthamiana* epidermal cells expressing GFP-LcGLO1. Fluorescence in epidermal cells was detected by confocal microscopy 48 hpa. The RFP-tagged PTS1 was used as the peroxisomal marker. Scale bars, 20 μm. The fluorescence intensity charts on the right correspond respectively with the white arrow cross-sections in the images to their left. (B) Co-expressing PlAvh133-RFP and GFP-LcGLO1 was subjected to confocal imaging after plasmolysis. *N. benthamiana* leaves were treated with 0.7 mol/L mannitol for plasmolysis. Fluorescent signals were observed at 36 hpa. Triangles indicate Hechtian strands, arrows indicate the PM region, and asterisks indicate the plant cell wall. Bars = 20 μm. (C) Anti-RFP was used to detect the expression of RFP-PlAvh133, RFP-α1-helixM5, RFP-α1-helixM6, and RFP. Anti-GFP was used to detect the expression of GFP-LcGLO1 proteins in *N. benthamiana* leaves. Protein loading is indicated by Ponceau S staining. Experiments were repeated three times with similar results.


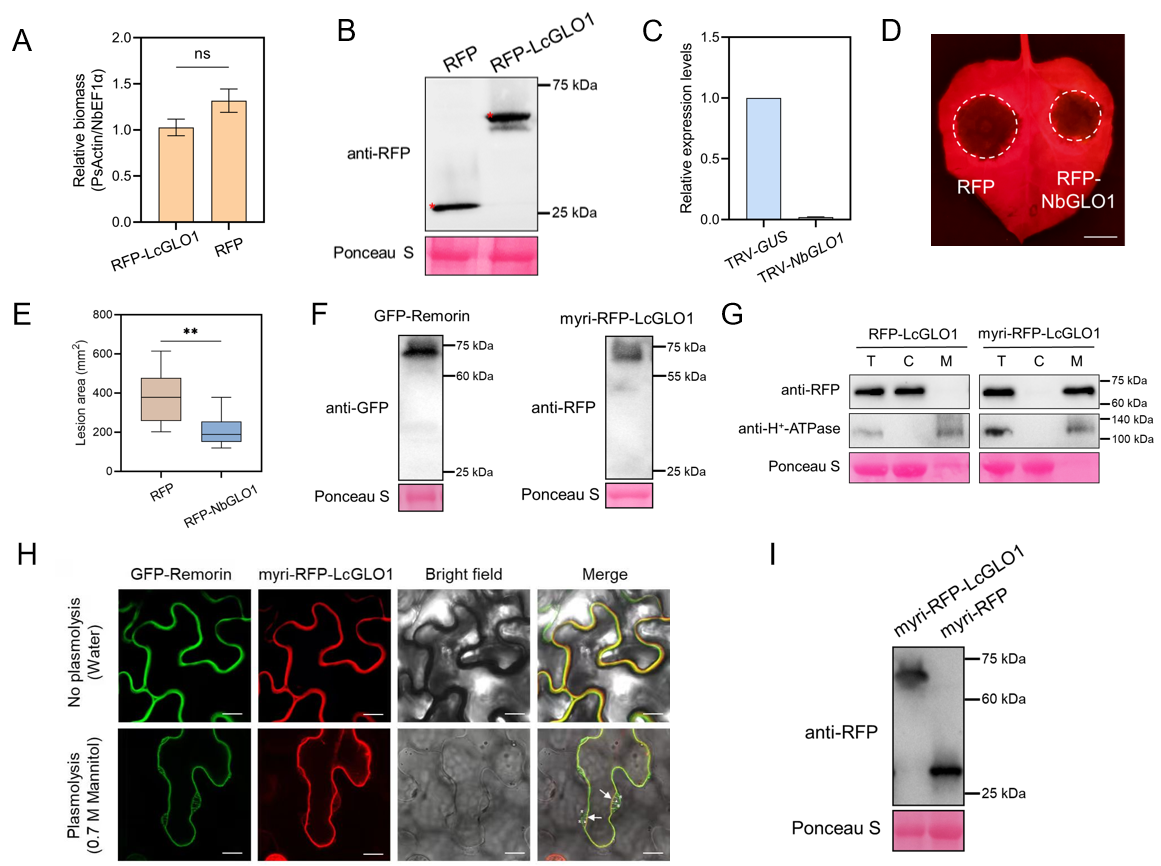


**Supplemental figure 8. Silencing of *NbGLO1* in *N. benthamiana* and the infection assays on NbGLO1-expressing plants.** (A) *N. benthamiana* leaves transiently expressing RFP-LcGLO1 did not significantly reduce the biomass of *P. capsici*. The relative biomass of *P. capsici* was measured at 48 hours post-inoculation (hpi) using qPCR. Data represent means ± SD. ns indicate no significant difference. (B) Anti-RFP was used to detect the expression of RFP-LcGLO1 and RFP proteins. Protein loading is indicated by Ponceau S staining. Expected protein bands are indicated by red asterisks. Experiments were repeated three times with similar results. (C) The relative expression levels of *NbGLO1* in *NbGLO1*-silenced plants. The relative expression level was normalized to that of *GUS* control. The constitutively expressed gene *NbEF1α* was used as internal reference. Data represent means ± SD. Experiments were repeated three times with similar results. (D and E) NbGLO1 contributed to resistance against *P. capsici*. *N. benthamiana* leaves expressing RFP-NbGLO1 or RFP were inoculated with *P. capsici*. Lesion development was measured and photographed at 48 hpi. Scale bar, 1 cm. Asterisks indicate significant differences. **p < 0.01; Student’s t-test; n=27. The central horizontal line denotes the median; vertical box height corresponds to interquartile range, respectively; and the whiskers show the maximum and minimum values within the analyzed dataset. (F and I) Anti-RFP and Anti-GFP ware used to detect the expression of GFP-Remorin, myri-RFP and myri-RFP-LcGLO1 proteins in *N. benthamiana* leaves. Protein loading is indicated by Ponceau S staining. Experiments were repeated three times with similar results. (G) Subcellular fractionation of extracts from *N. benthamiana* leaves expressing myri-RFP-LcGLO1. T, total protein; C, cytosolic fraction; M, microsomal fraction. (H) myri-RFP-LcGLO1 and GFP-Remorin ware subjected to confocal imaging after plasmolysis. *N. benthamiana* leaves were treated with 0.7 mol/L mannitol for plasmolysis. Fluorescent signals were observed at 36 hpa. Triangles indicate Hechtian strands, arrows indicate the PM region, and asterisks indicate the plant cell wall. Bars = 20 μm.


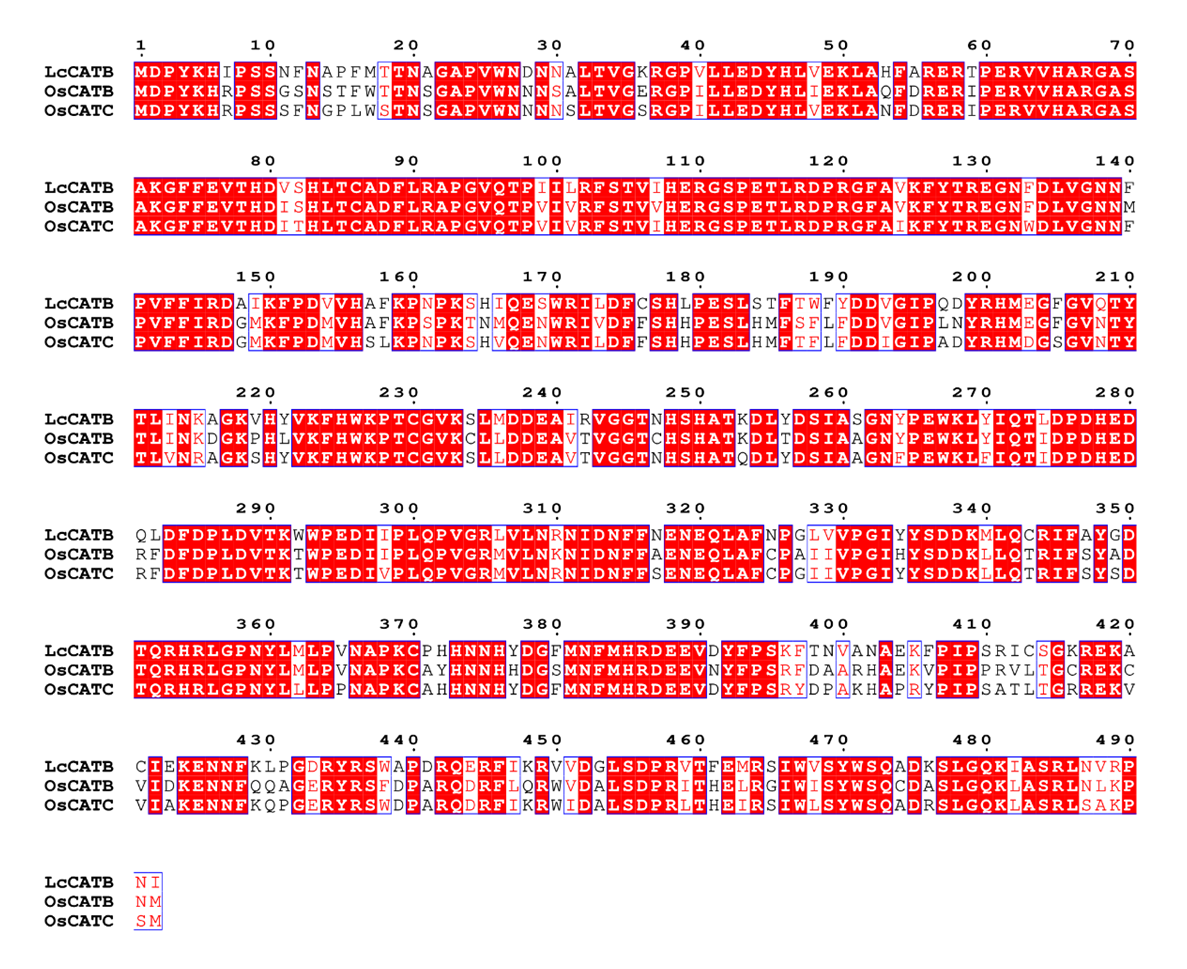


**Supplemental figure 9. Protein sequence alignment of LcCATB, OsCATB and OsCATC.** The sequence alignment was generated in Clustal W, and fully conserved sites are shown with red background, while sites with red font have similar amino acids.


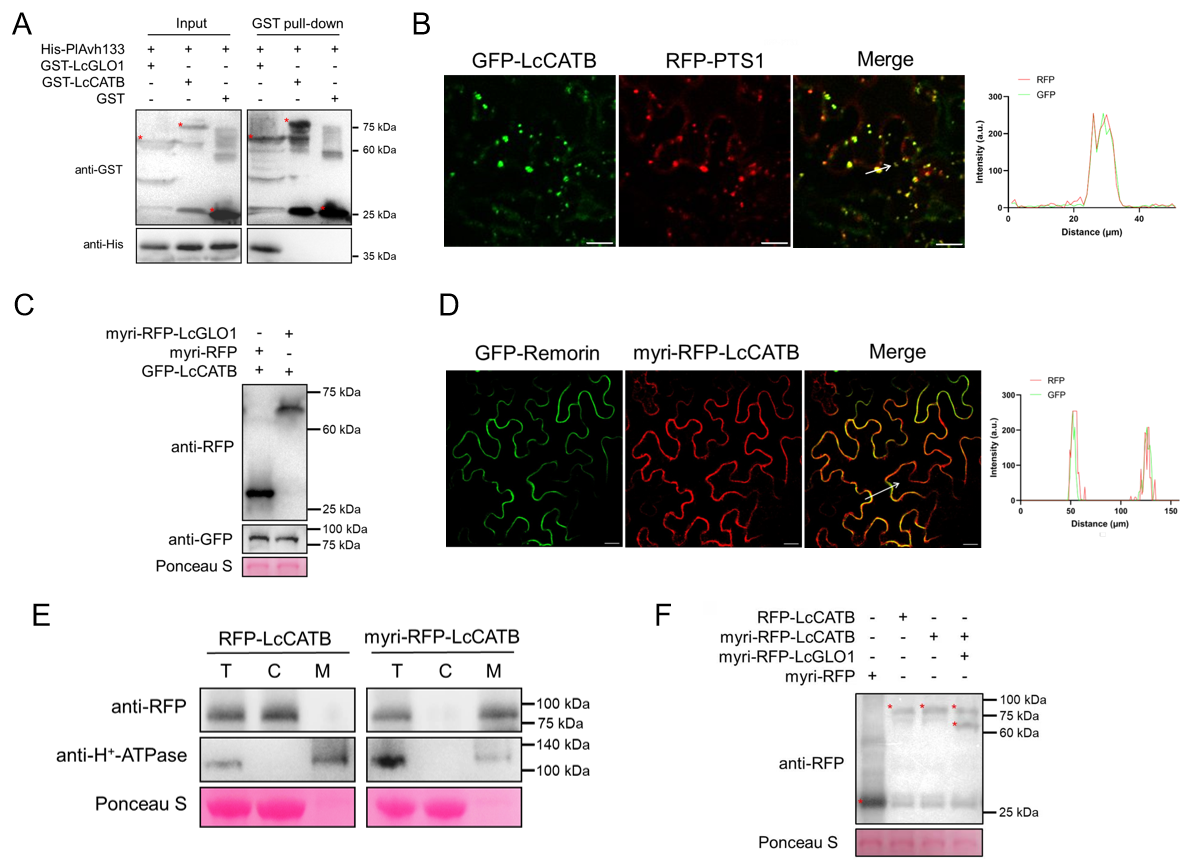


**Supplemental figure 10. Subcellular localization of LcCATB and PM-localized LcCATB.** (A) In vitro GST pull-down of His-PlAvh133 and GST-LcCATB**.** His-PlAvh133 and GST-LcCATB were expressed in *E. coli*. Co-precipitation of His-PlAvh133 with GST-LcCATB was examined by Western blotting before (input) and after affinity purification (pull-down) using glutathione agarose beads. GST-LcGLO1 and GST were used as the positive and negative control, respectively. In Western blot assays, expected protein bands are indicated by red asterisks. The experiments were performed three times with similar results. (B and D) Confocal images of *N. benthamiana* epidermal cells expressing GFP-LcCATB or myri-RFP-LcCATB. Fluorescence in epidermal cells was detected by confocal microscopy 48 hpa. The RFP-tagged PTS1 and GFP-tagged Remorin were used as the peroxisomal and PM marker, respectively. Scale bars, 20 μm. The fluorescence intensity charts on the right correspond respectively with the white arrow cross-sections in the images to their left. (C and F) Anti-RFP and Anti-GFP ware used to detect the expression of GFP-LcCATB, RFP-LcCATB, myri-RFP-LcCATB, myri-RFP-LcGLO1 and myri-RFP proteins in *N. benthamiana* leaves. Protein loading is indicated by Ponceau S staining. Expected protein bands are indicated by red asterisks. Experiments were repeated three times with similar results. (E) Subcellular fractionation of extracts from *N. benthamiana* leaves expressing myri-RFP-LcCATB. T, total protein; C, cytosolic fraction; M, microsomal fraction.


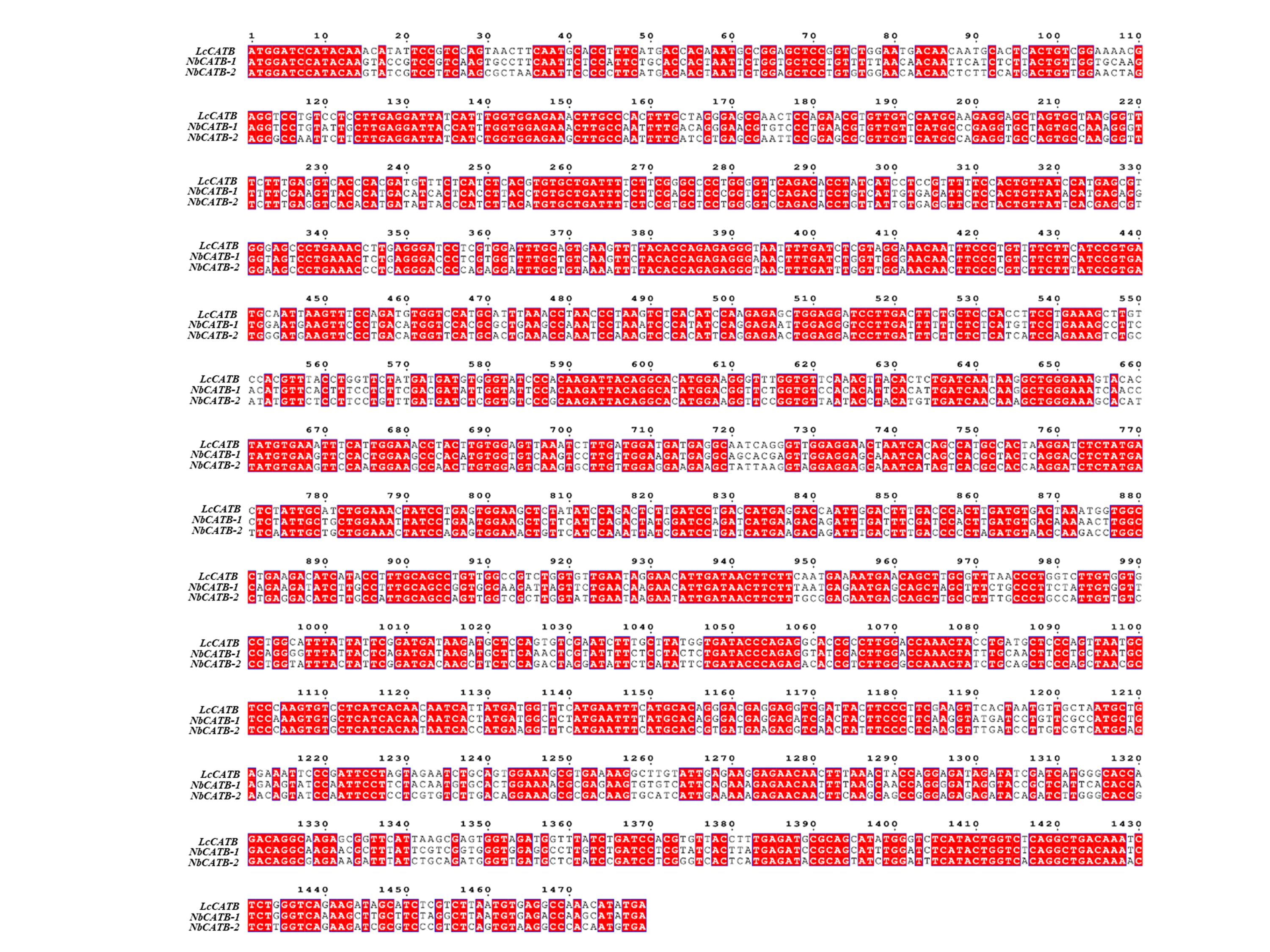


**Supplemental figure 11. Nucleic acid sequence alignment of *LcCATB*, *NbCATB-1* and *NbCATB-2*.** The sequence alignment was generated in Clustal W, and fully conserved sites are shown with red background.


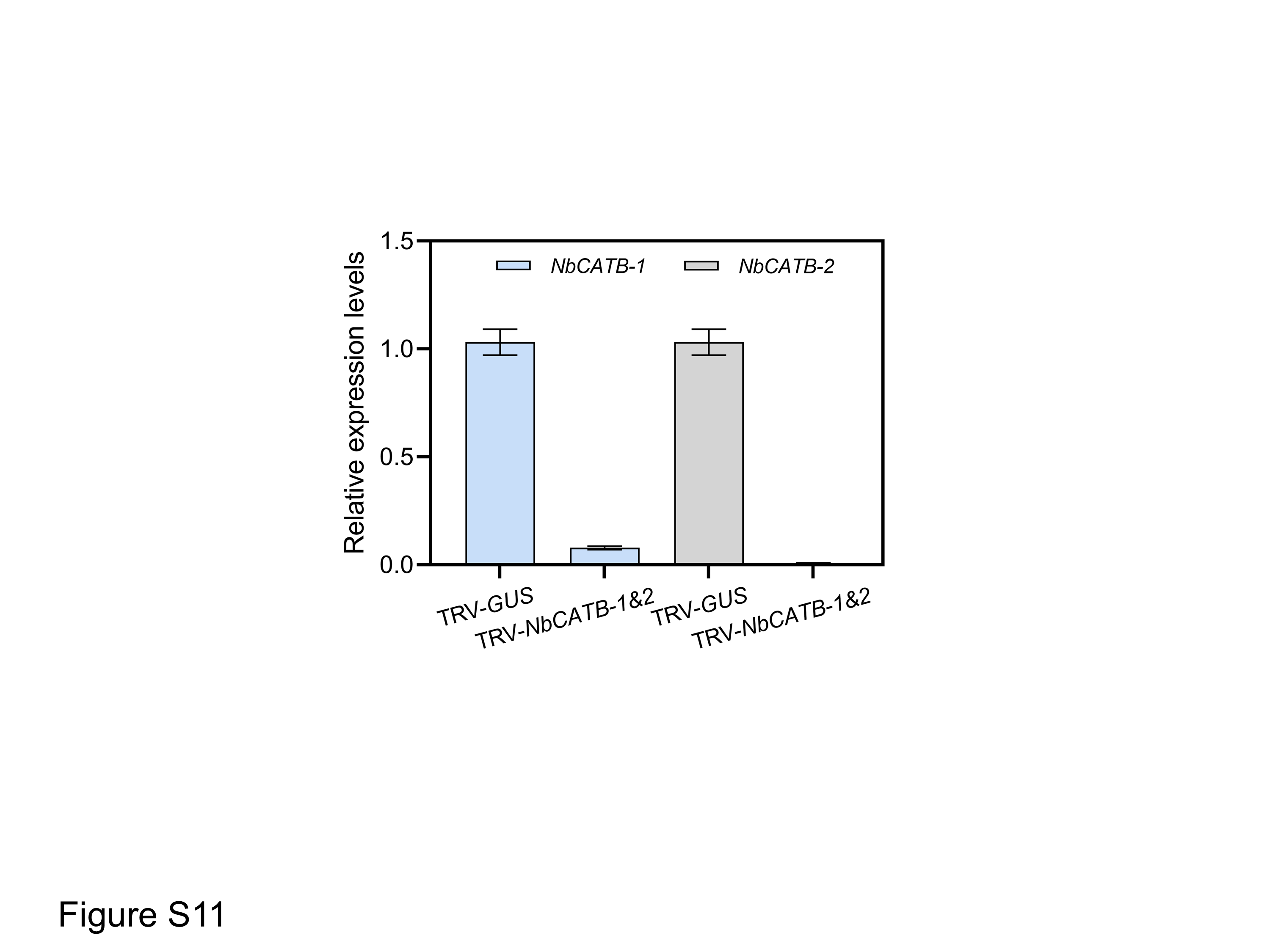


**Supplemental figure 12. Relative transcript levels of NbCATB-1 and NbCATB-2 in NbCATB-silenced *N. benthamiana* plants.** The relative expression level was normalized to that of *GUS* control. The constitutively expressed gene *NbEF1α* was used as internal reference. Data represent means ± SD. Experiments were repeated three times with similar results.


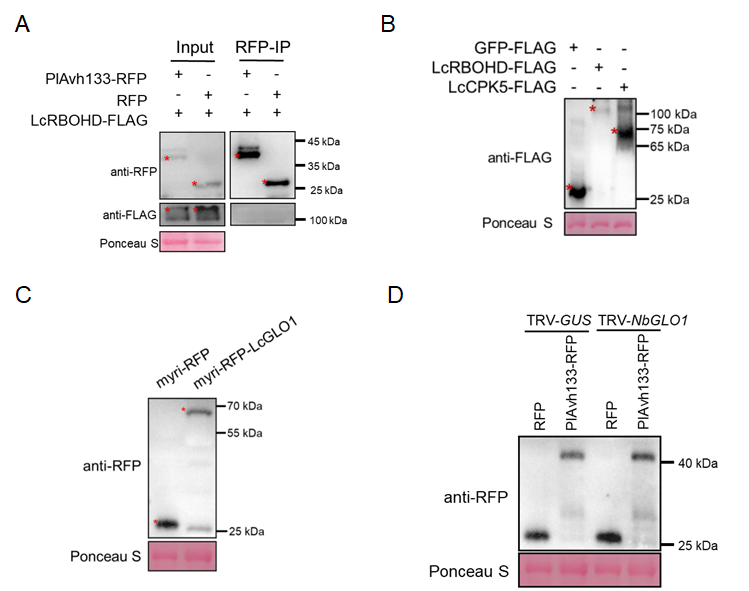


**Supplemental figure 13. PlAvh133 does not directly interact with LcRBOHD.** (A) *In vivo* co-IP of PlAvh133 with LcRBOHD. Total proteins were extracted from *N. benthamiana* leaves expressing PlAvh133-RFP or RFP (control) together with LcRBOHD-FLAG. The immune complexes were pulled down using anti-RFP agarose beads. Protein loading is indicated by Ponceau S staining. (B-D) Anti-RFP and Anti-FLAG were used to detect the expression of GFP-FLAG, LcRBOHD-FLAG, LcCPK5-FLAG, PlAvh133-RFP, myri-RFP-LcGLO1, RFP and myri-RFP proteins in *N. benthamiana* leaves. Protein loading is indicated by Ponceau S staining. Expected protein bands are indicated by red asterisks. Experiments were repeated three times with similar results.


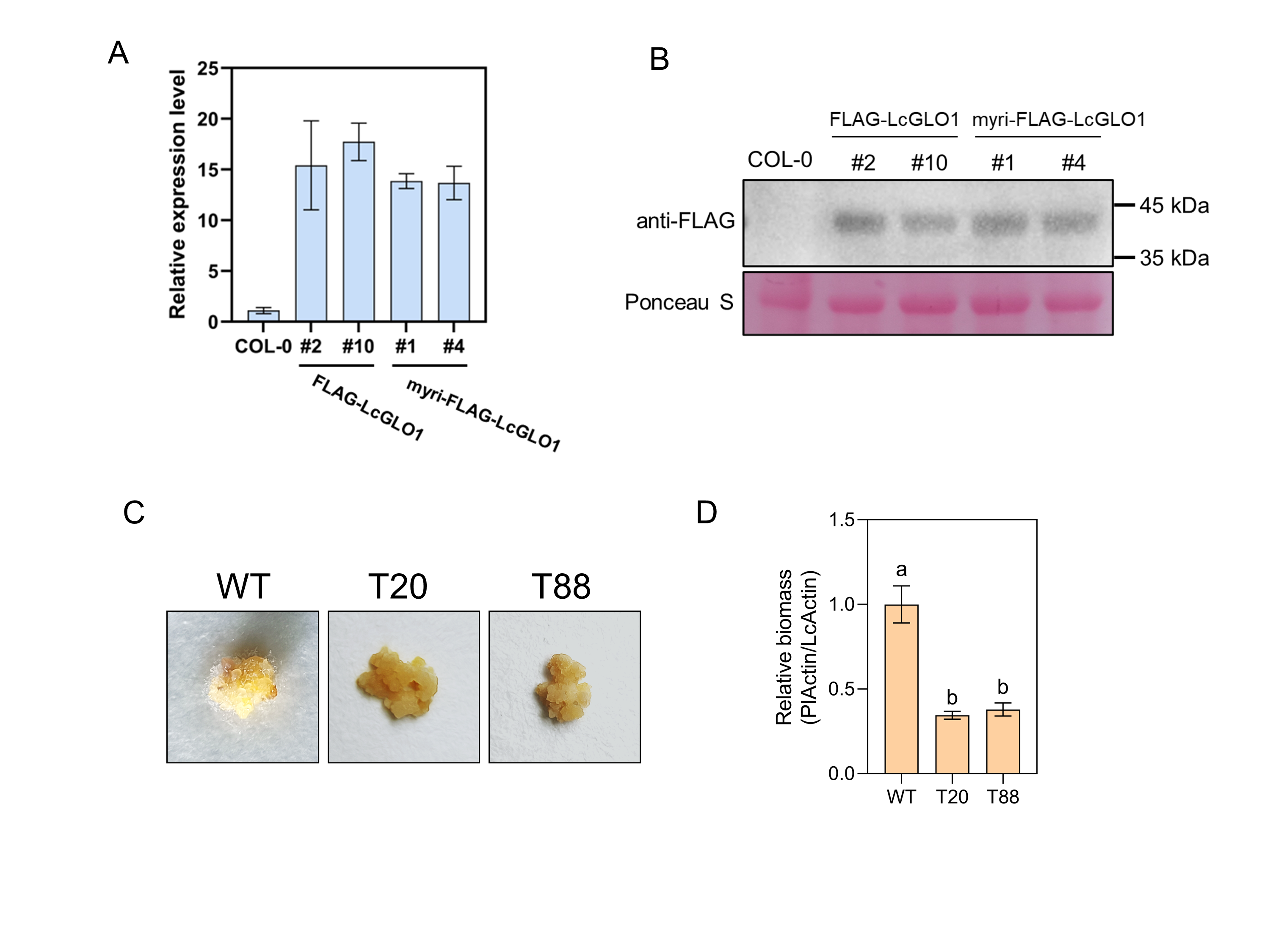


**Supplemental figure 14. Overexpression of** **FLAG-LcGLO1 and myri-FLAG-LcGLO1 in Arabidopsis and infection assay of the *P. litchii* PlAvh133-overexpressing mutants on litchi callus.** (A) Relative transcript levels of FLAG-LcGLO1 and myri-FLAG-LcGLO1 were determined by qRT-PCR in the respective transgenic Arabidopsis plants. The relative expression levels were calibrated to WT set as 1. The constitutively expressed gene, *AtUBC9*, was used as internal reference. (B) Anti-FLAG were used to detect the expression of FLAG-LcGLO1 and myri-FLAG-LcGLO1 proteins in transgenic Arabidopsis plants. Protein loading is indicated by Ponceau S staining. Experiments were repeated three times with similar results. (C and D) *P. litchii* *PlAvh133* knockout mutants exhibited reduced virulence to litchi calli. Litchi calli were inoculated with zoospore suspensions from *P. litchii* WT strain SHS3 or *PlAvh133* knockout mutants (T20 and T88). Disease symptoms were photographed at 72 hpi, and relative biomass of *P. litchii* was determined by qPCR. Data represent means ± SD. Experiments were repeated three times with similar results. Different letters indicate significant differences using Duncan’ s multiple range test at p<0.01.

**Supplemental Table 1 *Nicotiana benthamiana* proteins identified by CoIP/MS screen of *Peronophythora litchii* RXLR effector PlAvh133**

| **Accession (Sol Genomics Network)** | **Description** | **Peptide spectrum matches** |
| --- | --- | --- |
| Niben101Scf05368g03015.1 | Ribulose bisphosphate carboxylase/oxygenase activase | 26 |
| Niben101Scf06349g00008.1 | Carbonic anhydrase | 10 |
| Niben101Scf04099g03003.1 | Elongation factor Tu | 10 |
| Niben101Scf05536g01013.1 | Fructose-bisphosphate aldolase | 7 |
| Niben101Scf12308g00007.1 | ATP synthase subunit beta | 6 |
| Niben101Scf06898g00001.1 | Tubulin beta chain | 6 |
| Niben101Scf02937g04001.1 | Glutamate--glyoxylate aminotransferase | 6 |
| Niben101Scf01497g03008.1 | Catalase | 6 |
| Niben101Scf03275g00016.1 | Tubulin alpha chain | 4 |
| Niben101Scf05438g05014.1 | Aminomethyltransferase | 4 |
| Niben101Scf04886g05003.1 | Heat shock cognate 70 | 4 |
| Niben101Scf04174g05001.1 | Peroxisomal (S)-2-hydroxy-acid oxidase | 3 |
| Niben101Scf06590g00003.1 | L-ascorbate peroxidase 2 | 2 |
| Niben101Scf08020g01012.1 | 2-Cys peroxiredoxin | 2 |
| Niben101Scf01777g03022.1 | Elongation factor 2-like | 2 |
| Niben101Scf02030g02010.1 | Coatomer subunit beta | 2 |
| Niben101Scf08020g01012.1 | 2-Cys peroxiredoxin BAS1 | 2 |

Note: only the *N. benthamiana* proteins identified by having at least two peptide matches with equal or peptide matches in PlAvh133 treatment are more than GFP control are reported in this table.

**Supplemental Table 2 Primers used in this study**

| **Primer names** | **Sequences (5'-3')** | **Purpose** |
| --- | --- | --- |
| q133-F | CCTCCACAGTCGTGGAAAAT | qRT-PCR |
| q133-R | GGCGTAGCTGCTTTATCGTC | qRT-PCR |
| qPlActin-F | TCACGCTATTGTTCGTCTGG | qRT-PCR |
| qPlActin-R | TCATCTCCTGGTCAGAGTCC | qRT-PCR |
| qLcActin-F | ACCGTATGAGCAAGGAAATCACTG | qRT-PCR |
| qLcActin-R | TCGTCGTACTCACCCTTTGAAATC | qRT-PCR |
| qNbEF1α-F | TTGCTTGCTTTCACCCTTGG | qRT-PCR |
| qNbEF1α-R | TCGAAACCAGAGATGGGGAC | qRT-PCR |
| qLcGLO1-F | GCTGAAAGGGCTGGTTTCAAG | qRT-PCR |
| qLcGLO1-R | GCCAGCGACATATGAAGCAAG | qRT-PCR |
| qNbCATB-1-F | TACAAGTACCGTCCGTCAAGTG | qRT-PCR |
| qNbCATB-1-R | AAGCAATACAGGACCTCTTGCA | qRT-PCR |
| qNbCATB-2-F | GGAGGTGATCTGATCATTGCGA | qRT-PCR |
| qNbCATB-2-R | ACTATCCAAACAAGCTGTTTATGCA | qRT-PCR |
| TRV2-NbGLO1-F | ATTCTGTGAGTAAGGTTACCGAATTCAGGATGTTCAGTGGCTCCAGACTA | Constructtion TRV2 vector for VIGS in *N. benthamiana* |
| TRV2-NbGLO1-R | CCCCATGGAGGCCTTCTAGAGAATTCAGAAAACTACTGGCCTTCCAATAA | Constructtion TRV2 vector for VIGS in *N. benthamiana* |
| TRV2-NbCATB-F | GTGAGTAAGGTTACCGAATTCGAGGAGATCGACTACTTCCCT | Constructtion TRV2 vector for VIGS in *N. benthamiana* |
| TRV2-NbCATB-R | TGGAGGCCTTCTAGAGAATTCGTATGAGATCCAAATGCTGC | Constructtion TRV2 vector for VIGS in *N. benthamiana* |
| TRV2-F | TTATGTTCAGGCGGTTCTT | Verifing primers used in TRV2 |
| TRV2-R | CTTCAGACACGGATCTACTT | Verifing primers used in TRV2 |
| pBINGFP2-F | GAACCCTAATTCCCTTATCTG | Verifing primers used in pBinGFP2 |
| pBINGFP2-R | AAGACCCCAACGAGAAGC | Verifing primers used in pBinGFP2 |
| pBINFLAG-F | ACACAACATATACAAAACAAACG | Verifing primers used in pBINFLAG |
| pBINFLAG-R | ACCATGATTACGCCAAGCTG | Verifing primers used in pBINFLAG |
| pBINGFP133-F | CTGTACAAGGGTACCCCCGGGATGAGTAGCGACACTCTCTCCGCTACCG | Construction pBINGFP2::PlAvh133 |
| pBINGFP133-R | AGAGGATCCGTCGACCCCGGGTTAGTCCACAGCGCGCACGTAGTCGATG | Construction pBINGFP2::PlAvh133 |
| pBINRFP133-F | GCCGGTACCCCCGGGATGAGTAGCGACACTCTCTCC | Construction pBINRFP::PlAvh133 |
| pBINRFP133-R | GGAGGCCATCCCGGGGTCCACAGCGCGCACGTA | Construction pBINRFP::PlAvh133 |
| pBINFLAG133-F | GCCGGTACCCCCGGG AGTAGCGACACTCTCTCC | Construction pBINFLAG::PlAvh133 |
| pBINFLAG133-R | TTTGTAGTCCCCGGGGTCCACAGCGCGCA | Construction pBINFLAG::PlAvh133 |
| 133nluc-F | ACGGGGGACGAGCTCGGTACCATGAGTAGCGACACTCTCTCCGC | Construction pCAMBIA1300-nLUC::PlAvh133 |
| 133nluc-R | CGCGTACGAGATCTGGTCGACGTCCACAGCGCGCACGTAGT | Construction pCAMBIA1300-nLUC::PlAvh133 |
| 133sgRNA-sense | GAATTTGCCCTCCACAGTCGTGG | Constructtion pYF2.3G-RibosgRNA to target PlAvh133 |
| 133sgRNA-antisense | ATTCGACTGCTTATCAACTGCGG | Constructtion pYF2.3G-RibosgRNA to target PlAvh133 |
| RPL41_Pseq_F | CAAGCCTCACTTTCTGCTGACTG | Verifing primers used in pYF2.3G-RibosgRNA |
| M13-F2 | GTTGTAAAACGACGGCCAGT | Verifing primers used in pYF2.3G-RibosgRNA |
| M13-F | TGTAAAACGACGGCCAGT | Verifing primers used in knockout vector |
| M13-R | CAGGAAACAGCTATGACC | Verifing primers used in knockout vector |
| NPTII-F | ATGATTGAACAAGATGGATT | Generation vector to knockout *PlAvh133* in *P. litchii* |
| NPTII-R | TCAGAAGAACTCGTCAAGAA | Generation vector to knockout *PlAvh133* in *P. litchii* |
| Avh133-F1 | AGCTAGTGGGCACGGGTGGAA | Primers used in verifing PlAvh133 knockout mutants in *P. litchii* |
| Avh133-R1 | CACGAGTTCGTCTGTGAAAGG | Primers used in verifing PlAvh133 knockout mutants in *P. litchii* |
| Avh133-OE-F | CTCGAGGTCGACGGTATCGATATGCGCAAGTGCATTCTTCTCCT | Constructtion pTORmRFP4 vector for overexpression PlAvh133 in *P. litchii* |
| Avh133-OE-R | CTCGGAGGAGGCCATCGTACGGTCCACAGCGCGCACGTAGTCGA | Constructtion pTORmRFP4 vector for overexpression PlAvh133 in *P. litchii* |
| pTOR-F | TCACTCTCACGTGCCCAAGTCC | Verifing primers used in pTORmRFP4 |
| pTOR-R | TTGTATTAAATGCATAGACACA | Verifing primers used in pTORmRFP4 |
| pet32a_133F | GCTGATATCGGATCC ATGAGTAGCGACACTCTCTCC | Construction pET32a::PlAvh133 |
| pet32a_133R | CTCGAATTCGGATCCTTAGTCCACAGCGCGCAC | Construction pET32a::PlAvh133 |
| pGEXLcGLO1_F | GGATCCCCGGAATTC ATGGAGGAGATAACAAAT | Construction pGEX6P1::LcGLO1 |
| pGEXLcGLO1_R | TCGACCCGGGAATTCTTATAACCTGGCTACAGC | Construction pGEX6P1::LcGLO1 |
| LcGLO1_clucF | GCGTCCCGGGGCGGTACCATGGAGGAGATAACAAAT | Construction pCAMBIA1300-cLUC::LcGLO1 |
| LcGLO1_clucR | TGTTGGATCCCGGGTACCTTATAACCTGGCTACAGCGT | Construction pCAMBIA1300-cLUC::LcGLO1 |
| pBINFLAGLcGLO1-F | GCCGGTACCCCCGGG ATGGAGGAGATAACAAATG | Construction pBINFLAG::LcGLO1 |
| pBINFLAGLcGLO1-R | TTTGTAGTCCCCGGGTAACCTGGCTACAGCG | Construction pBINFLAG::LcGLO1 |
| RFP_NbREM_F | ACGATAGCCGGTACCCCCGGG ATGGCAGAAGTAGAAGCTACG | Construction pBINGFP2:: Remorin |
| RFP_NbREM_R | CTCGGAGGAGGCCATCCCGGGAAAACATCCAAGGAGTTTCTTTG | Construction pBINGFP2:: Remorin |
| GFP_NbREM_F | CTGTACAAGGGTACCCCCGGGATGGCAGAAGTAGAAGCTACG | Construction pBINRFP:: Remorin |
| GFP_NbREM_R | AGAGGATCCGTCGACCCCGGGAAAACATCCAAGGAGTTTCTTTG | Construction pBINRFP:: Remorin |
| pCAMLcGLO1-F | CACCACCACCACGTG ATGGAGGAGATAACAAATGT | Construction pCAMBIA1302::LcGLO1 |
| pCAMLcGLO1-R | GGGGAAATTCGAGCTGGTCACCTTATAACCTGGCTACAGC | Construction pCAMBIA1302::LcGLO1 |
| pBINRFP-F | ACCATCGTGGAACAGTACGA | Verifing primers used in pBINRFP-R |
| pBINRFP-R | ACCATGATTACGCCAAGCTG | Verifing primers used in pBINRFP-R |
| myriRFP-F | TTACGAACGATAGCCGGTACCATGGGAATTTGTATGTCTAGAATGGCCTCCTCCGAGGAC | Construction pBIN::myriRFP |
| myriRFP-R | GCGGACTCTAGTTCATCTAGAGGCGCCGGTGGAGTGG | Construction pBIN::myriRFP |
| RFPLcGLO1-F | CACTCCACCGGCGCC GTCGACATGGAGGAGATAACAAAT | Construction pBINRFP::LcGLO1 |
| RFPLcGLO1-R | TCTAGATCAGGATCCGTCGACTTATAACCTGGCTACAGCG | Construction pBINRFP::LcGLO1 |
| myriRFPLcGLO1-F | CACTCCACCGGCGCCTCTAGA ATGGAGGAGATAACAAATGT | Construction pBIN::myriRFPLcGLO1 |
| myriRFPLcGLO1-R | GCGGACTCTAGTTCATCTAGATTATAACCTGGCTACAGC | Construction pBIN::myriRFPLcGLO1 |
| RFPLcCATB-F | CACTCCACCGGCGCC GTCGACATGGATCCATACAAACATATTC | Construction pBINRFP::LcCATB |
| RFPLcCATB-R | TCATCTAGAGGATCCGTCGACTCATATGTTTGGCCTCACAT | Construction pBINRFP::LcCATB |
| myriRFPLcCATB-F | CACTCCACCGGCGCCGTCGACATGGGAATTTGTATGTCTAGAATGGATCCATACAAACATAT | Construction pBIN::myriRFPLcCATB |
| myriRFPLcCATB-R | GCGGACTCTAGTTCATCTAGATCATATGTTTGGCCTCACAT | Construction pBIN::myriRFPLcCATB |
| GFPLcCATB_F | CTGTACAAGGGTACCCCCGGG ATGGATCCATACAAACATATTC | Construction pBINGFP2::LcCATB |
| GFPLcCATB_R | AGAGGATCCGTCGACCCCGGGTCATATGTTTGGCCTCACAT | Construction pBINGFP2::LcCATB |
| FLAGAvh133-α1F | AGAGGCATGACGGAA GTCACTAACCCGATGC | Generation PlAvh133 α-helix deletion mutants |
| FLAGAvh133-α1R | GCATCGGGTTAGTGACTTCCGTCATGCCTCT | Generation PlAvh133 α-helix deletion mutants |
| FLAGAvh133-α2F | AGACGTGGGTCACTAAC AAGGACGTTTCAAATATC | Generation PlAvh133 α-helix deletion mutants |
| FLAGAvh133-α2R | GATATTTGAAACGTCCTTGTTAGTGACCCACGTCT | Generation PlAvh133 α-helix deletion mutants |
| FLAGAvh133-α3F | TCAAGAAGGACGTTTCAAAGCAGGTCACCCTG | Generation PlAvh133 α-helix deletion mutants |
| FLAGAvh133-α3R | CAGGGTGACCTGCTTTGAAACGTCCTTCTTGA | Generation PlAvh133 α-helix deletion mutants |
| FLAGAvh133-α4F | CGGAAGCAGGTCACCTTGAAGTTGGACCCGGA | Generation PlAvh133 α-helix deletion mutants |
| FLAGAvh133-α4R | TCCGGGTCCAACTTCAAGGTGACCTGCTTCCG | Generation PlAvh133 α-helix deletion mutants |
| FLAGAvh133-α5F | TCCACGCTGGCTCAAACCCCGGATTGGC | Generation PlAvh133 α-helix deletion mutants |
| FLAGAvh133-α5R | GCCAATCCGGGGTTTGAGCCAGCGTGGA | Generation PlAvh133 α-helix deletion mutants |
| FLAGAvh133-α6R | TTTGTAGTCCCCGGGGTCCACAGCGCGCACTCCGGGGTTCTTTGC | Generation PlAvh133 α-helix deletion mutants |
| FLAG/RFPAvh133-αF | GCCGGTACCCCCGGG ATGAGTAGCGACACTCTCTCC | Generation PlAvh133 α-helix deletion mutants |
| RFPAvh133-αR | GGAGGCCATCCCGGGGTCCACAGCGCGCACGTA | Generation PlAvh133 α-helix deletion mutants |
